# Unravelling 3D Dynamics and Hydrodynamics during Incorporation of Dielectric Particles to an Optical Trapping Site

**DOI:** 10.1021/acsnano.2c11753

**Published:** 2023-02-17

**Authors:** Boris Louis, Chih-Hao Huang, Rafael Camacho, Ivan G. Scheblykin, Teruki Sugiyama, Tetsuhiro Kudo, Marc Melendez, Rafael Delgado-Buscalioni, Hiroshi Masuhara, Johan Hofkens, Roger Bresoli-Obach

**Affiliations:** † Molecular Imaging and Photonics, Department of Chemistry, KU Leuven, Celestijnenlaan 200F, Leuven 3001, Belgium; ‡ Department of Applied Chemistry, National Yang Ming Chiao Tung University, 1001 Ta Hsueh Road, Hsinchu 300093, Taiwan; § Center for Cellular Imaging, Core Facilities, the Sahlgrenska Academy, University of Gothenburg, Medicinaregatan 5A-7A, Box 413, Gothenburg 40530, Sweden; ∥ Division of Chemical Physics and NanoLund, Lund University, Kemicentrum Naturvetarvägen 16, P.O. Box 124, Lund 22100, Sweden; ⊥ Division of Materials Science, Nara Institute of Science and Technology, 8916-5 Takayamacho, Ikoma, Nara 630-0101, Japan; # Departamento de Física Teórica de la Materia Condensada, Institut for Condensed Matter (IFIMAC), Universidad Autónoma de Madrid, Campus de Cantoblanco, Madrid 28049, Spain; ¶ Center for Emergent Functional Matter Science, National Yang Ming Chiao Tung University, 1001 Ta Hsueh Road, Hsinchu 300093, Taiwan; □ Max Planck Institute for Polymer Research, Ackermannweg 10, Mainz 55128, Germany; ▼ AppLightChem, Institut Químic de Sarrià, Universitat Ramon Llull, Via Augusta 390, Barcelona, Catalunya 08017, Spain

**Keywords:** optical trapping, multiplane widefield microscopy, hydrodynamics, optical field, particle tracking, 3D imaging

## Abstract

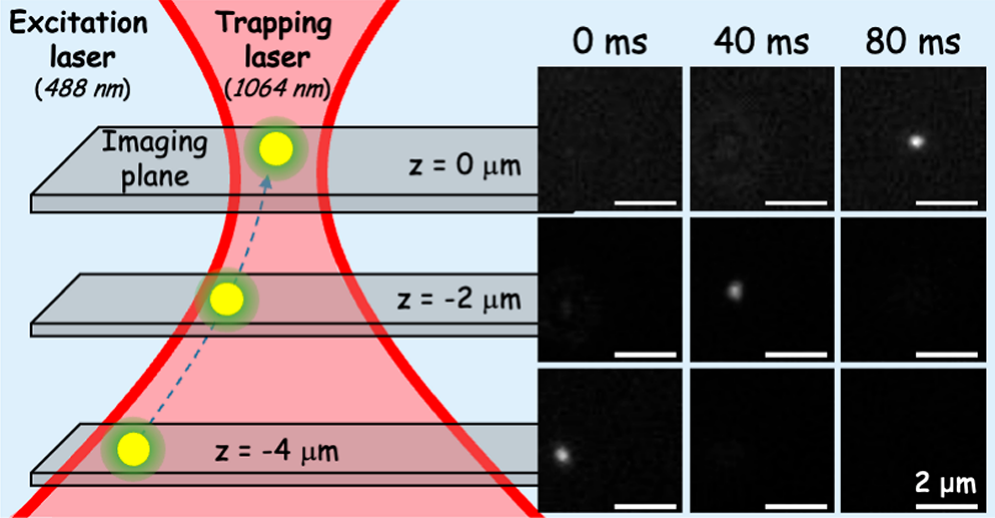

Mapping of the spatial and temporal motion of particles inside an optical field is critical for understanding and further improvement of the 3D spatio-temporal control over their optical trapping dynamics. However, it is not trivial to capture the 3D motion, and most imaging systems only capture a 2D projection of the 3D motion, in which the information about the axial movement is not directly available. In this work, we resolve the 3D incorporation trajectories of 200 nm fluorescent polystyrene particles in an optical trapping site under different optical experimental conditions using a recently developed widefield multiplane microscope (imaging volume of 50 × 50 × 4 μm^3^). The particles are gathered at the focus following some preferential 3D channels that show a shallow cone distribution. We demonstrate that the radial and the axial flow speed components depend on the axial distance from the focus, which is directly related to the scattering/gradient optical forces. While particle velocities and trajectories are mainly determined by the trapping laser profile, they cannot be completely explained without considering collective effects resulting from hydrodynamic forces.

## 1 Introduction

In 1986, Ashkin and co-workers proposed the use of tightly focused laser beams to manipulate individual microscale objects, a method now referred to as optical trapping or optical tweezers.^1^ In their initial work, they were capable of optically trapping and controlling polystyrene (PS) microparticles. Since then, optical trapping has been widely used in biology, chemistry, physics, and materials science.^2−9^ Optical trapping allows the user to gain control over a wide range of materials (e.g., metallic and dielectric particles, cells, bacteria, viruses, proteins, and even small molecules such as amino acids) and works under different optical conditions (e.g., different laser polarization, beam shape, or medium).^10−13^ Indeed, optical trapping is a powerful tool for the controlled fabrication and assembly of micro- and nanostructures in specific 2D/3D arrangements. Specifically, the different building blocks can be positioned by the optical force while the particle linkage is achieved using different approaches, such as biochemical binding, photopolymerization, or engineering of the physical interparticle colloidal forces.^14−17^ At interfaces, these structures can expand far away from the focus without the need of particle linkage, thereby generating up to submillimeter-sized optical matter.^18−21^ Lastly, it has been successfully employed to study particle motion and measure small forces (pN) at the nanoscale, which is relevant for a vast variety of biological processes.^22,23^

To generate an optical trap, a high flux of photons focused on a small area is required. Generally, this is achieved by tightly focusing a laser beam with a microscope objective lens with a high magnification and high numerical aperture (NA).^24^ The objective lens focuses the laser beam to a diffraction-limited focal spot, thereby producing strong optical gradients. When a particle enters the generated optical field, it will mainly experience two optical forces because of the interaction between the particle and the field. The scattering force is due to the momentum transfer from the photons to the particle via scattering and pushes the object along the light propagation pathway. Conversely, the gradient force is caused by the spatial intensity gradient of the laser beam, which drags the particles toward the position where the laser intensity is the largest. Considering the optical forces, a stable trapping spot can only be achieved when the induced gradient force is larger than the scattering force. Specifically, a large gradient/scattering force ratio is obtained by trapping dielectric particles using a large NA objective. Under such condition, the 3D position of the trapped particles can be controlled with high spatio-temporal precision.^3−6,10^ However, Brownian motion, as well as gravity forces, can affect the formation of a stable trapping spot.^25,26^ Therefore, a stable trapping spot can only be generated when the overall optical force is larger than other bespoke fluctuations.

A variety of fluorescence imaging techniques have been successfully combined with optical trapping, which range from widefield and total internal reflection fluorescence (TIRF)^27−29^ to more optically advanced techniques [confocal, stimulated emission depletion (STED), or stochastic optical reconstruction microscopy (STORM)].^30,31^ For instance, holographic optical tweezers have been used to immobilize and orient nonadherent cells, thereby enabling imaging sample sections with the direct STORM (dSTORM) super-resolved method.^32^ Other optical imaging strategies, such as transmission or darkfield microscopies, have also been used.^33^ However, these imaging techniques mainly record a 2D projection of a 3D image, or the third dimension needs to be scanned, which prevents the correct 3D visualization of dynamic samples. In addition, in most cases, optical trapping has only been used to immobilize the object, and no studies on the motion of the object neighboring the trapping site have been performed.

To achieve a more effective and selective manipulation of nano/micro-objects, it is essential to understand how the optical trapping dynamics changes as function of the optical properties of the laser beam and, in general, with the physicochemical properties of the surrounding environment. Furthermore, the scattering and gradient forces and the resulting hydrodynamics effects need to be studied in 3D.^34^ Indeed, when considering nanoparticles (NPs), any force induced on them will cause a movement of the NP in the fluid. Because of hydrodynamic effect, this force will be transmitted to the fluid and carried by viscous transport of momentum to other nearby NPs, thereby modifying their velocity according to the well-known Oseen tensor.^35^ By this collective mechanism, the spatially localized forces induced on the objects by the laser beam may potentially activate nonlinear terms in the NPs flow.^36^ It is known that above a certain threshold in the volume fraction of the NPs (about ϕ = 10^–4^) and driving energy (about 1 *k*_B_*T*/particle), hydrodynamic interactions between optically driven NPs can create interesting collective phenomena, such as swarming and superlinear scaling of the flow of NPs, with the laser power.^21,22,32^ It has to be mentioned that only a few works in optical trapping describe the kinetics of particle incorporation into and escaping from trapping sites.^37,38^ This is likely because the phenomenon occurs intrinsically in 3D, while standard setups are only able to acquire two-dimensional (*x*–*y*) data, which obtains a mere projection of the three-dimensional phenomenon.

**Figure 1:**
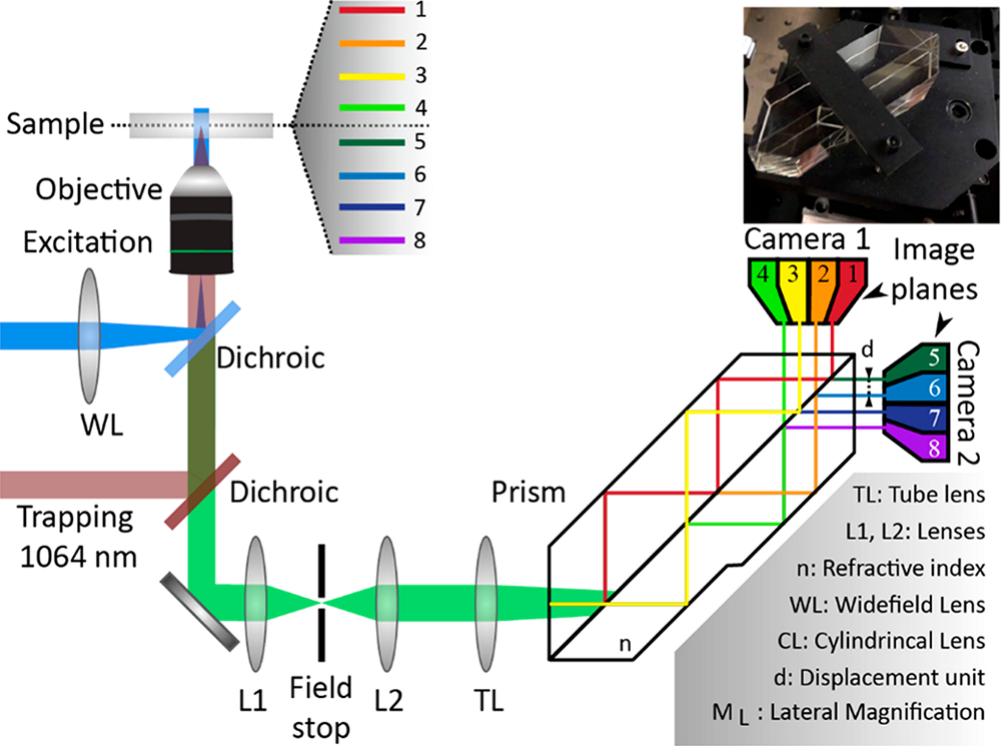
Schematic of the multiplane widefield microscope with an optical tweezer unit. The trapping laser and the proprietary prism are the two main components that differ from conventional widefield microscopes. The main axis of the prism acts as a beam splitter and splits the light 3 times, which results in 2^3^ (8) imaging planes, thereby yielding a total volume of 50 × 50 × 4 μm^3^ acquired simultaneously. The position of the planes was determined by calibration using a sample of 200 nm beads spin-casted on a coverslip (see Materials and Methods for a full description of the home-built multiplane widefield microscope).

Here, we used our developed widefield multiplane microscope to study the incorporation of individual 200 nm fluorescent PS NPs in 3D into the stable trapping site under a range of optical trapping conditions (e.g., laser polarization, laser power, and numerical aperture of the objective). Our multiplane imaging approach simultaneously acquires images from eight different depth planes, which yields a three-dimensional image (50 × 50 × 4 μm^3^; Figure 1) with fast acquisition rates (200 Hz).^39^ We could, therefore, follow the incorporation of the NPs into a trapping site with high 3D spatial and temporal resolution. We observed that the trapping laser profile plays a major role in the particle incorporation. We also observed interesting hydrodynamic effects, such as the formation of a preferential incorporation channel or nonlinear effects on the NP flows. As shown below, the 3D imaging technique presented in this paper allows uncovering of the signature of nonlinear hydrodynamic coupling.

## 2 Results and Discussion

### 2.1 Distribution, Flow Speed, and Optical Force of Nanoparticles at Multifocal Planes

Prior to investigation of the different optical conditions that influence the trapping, we studied the incorporation of 200 nm fluorescent PS NPs into a trapping site under a standard optical condition (36 mW, circularly polarized laser, NA 1.20, 60× water immersion objective). To obtain significant statistics, we analyzed 150 independent videos, which were recorded during the first five seconds after switching on the trapping laser. In each video, we observed one to four incorporation events. The trapping laser was focused on the first imaging plane (i.e., on top of the 50 × 50 × 4 μm^3^ image), which allowed us to follow the motion of particles up to a depth of 4 μm below the trapping site (Supplementary Video S1 and Figure S1, which contain selected frames showing the primary raw data obtained from the microscope). Even by visual inspection, we can easily observe the movement in the axial position of the NP during its incorporation to the trapping site.

**Figure 2:**
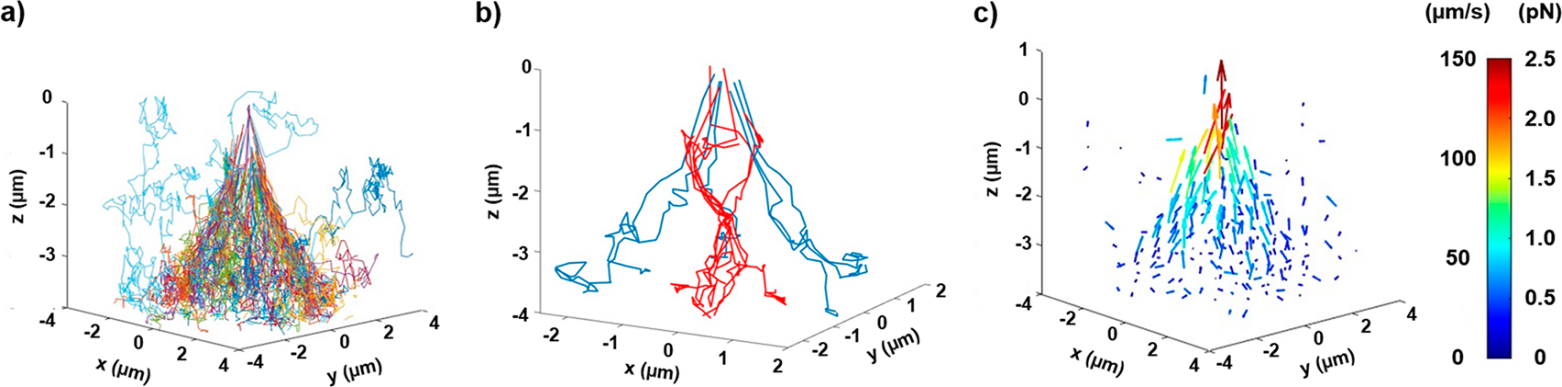
Incorporation of individual fluorescent nanoparticles in the trapping site. (a) 3D traces of all the trapping events acquired from 150 independent videos. The focal spot (i.e., trapping site) was localized approximately at *z* = 0 μm. (b) Representative incorporation trajectories going through the inner and outer cones (red and blue lines, respectively). (c) Distribution of 3D flow speed vectors for incorporation of the particles. The length of the arrow and its color denotes the magnitude of the speed/optical force vector.

Figure 2a shows all the detected 3D traces of NP incorporation into the trapping site (some of the particle traces located well outside the trapping domain have been removed for sake of clarity). At first glance, the incorporation of the NPs follows a shallow cone shape. Note that particles follow a Brownian motion outside the irradiated area and then exhibit a directional motion once they enter the “trapping region” (i.e., the high-intensity region of the optical field). The directional motion is related to the optical force, in which the incident photons transfer their momentum to the NPs to push them toward the focal spot (see Figure S2 for a representative trace example).^40^

From the trajectories, we observed that not all the particles go through a similar path. Indeed, some particles happened to be trapped for a few tenths of a millisecond in a metastable position a few micrometers below the optical trapping focus. Figure 2b shows example of such traces in red, while the blue trajectories present the type of path that particles generally take, following a cone shape. The presence of this metastable trapping position indicates that a local shallow minimum of the optical potential field is induced at that point. However, once the pushing–scattering force is strong enough, the NP can escape following the tightly focused laser propagation direction that points toward the external cone, where it finally reaches the focus.

**Figure 3:**
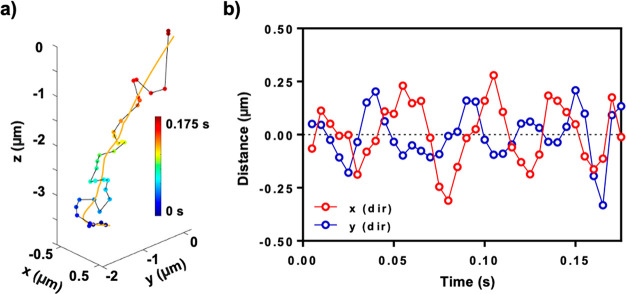
(a) A representative incorporation trace flowing through the external cone. As a visual aid (gold line), the trajectory is smoothed using a third-order Savitzky–Golay filter. The color scale denotes the time scale. (b) Relative distance between the previous trace and the smoothed trace in the *x* and *y* directions (red and blue lines, respectively).

Another peculiarity found upon a close inspection of the 3D trajectories is that the NPs flowing inside the cone do not follow a straight linear trajectory, but their motion is rather slightly modified, following a helicoidal trajectory with respect to the incoming axis (Figure 3a). The radius of the helicoidal trajectory is stochastic and is different for each NP. We note that in approximately half of the cases, it is hard to distinguish from random Brownian fluctuations. The optical force is directly related to the laser beam electric field,^41^ and such trajectories reflect the inner structure of the light beam.^42^ To investigate these helicoidal motions, we smoothed the oscillations of the incoming trajectory with a third-order Savitzky–Golay filter and calculated the distances between the smoothed and the tracked incoming helicoidal trajectories (Figure 3b). The distance between the smoothed and bare trajectories follows a periodic sinusoidal function along the *x* and *y* direction. The *x* direction is dephased roughly 90° with the *y* direction, which shows the helicoidal nature of the incorporation motion. The direction of the helicoidal trajectory (clockwise or anticlockwise) is stochastic for each incorporation event, which suggests that it is independent from the laser polarization.

To understand better the three-dimensional motions observed of the metastable trapping position and the helicoidal motion, we performed calculation of the tightly focused laser profile according to the angular spectrum representation theory for equivalent experimental optical conditions (Figures S3 and S4). The theoretical laser profile also revealed a laser power distribution with several annular rings due to the interference of the focusing beam, which effectively yielded a cone in 3D. Such a pattern is called a periodically converging pattern. Moreover, we note that the calculation shows the presence of a secondary focal point around 2 μm below the main focal point, which explains the observed metastable position. Finally, Figure S4 shows that the periodically converging pattern of the laser yields some sort of “zig-zag” in the intensity because of the succession of maxima and minima across the axial direction. It is clear that such changes in the direction of the gradient force will affect the trajectory and are sometimes likely the cause of the phenomenologically observed helicoidal trajectory. These results show the large potential of multiplane widefield microscopy to discover unknown phenomenon related to optical trapping.

Additional information about the incorporation phenomena can be obtained by analyzing the 3D flow speed distribution (Figure 2c). One can see that the direction of the particle motion follows the laser light propagation pathway, and its magnitude increases when the particles approach the focal spot up to about 150 μm/s under these experimental conditions.

We note that the experimental measurement of the NP velocity field provides indirect access to the optical force. At these small scales, the particle inertia is negligible; thus, the induced optical force can be estimated from the balance between the optical force and the viscous drag described by the Stokes drag force law (eq 1):^43^
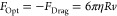
1where *F*_Opt_ and *F*_Drag_ are the optical and drag forces, respectively; η is the solvent dynamic viscosity; *R* is the particle radius; and *v* is the speed. Of note, eq 1 is only valid in the limit of an isolated NP in a very diluted solution, that is, when it is possible to neglect the hydrodynamic coupling occurring because of the perturbative flow created by neighboring NPs’ motion. Neglect of the hydrodynamic couplings between particles is possible in the dilute limit (a more rigorous approach would require complex simulations to solve the convolution of optical and hydrodynamic effects).^36^ Despite the fact that we observe evidence of hydrodynamic coupling (see below), the relatively small volume fraction used here indicates that eq 1 can be safely used to estimate the optical force field.

**Figure 4:**
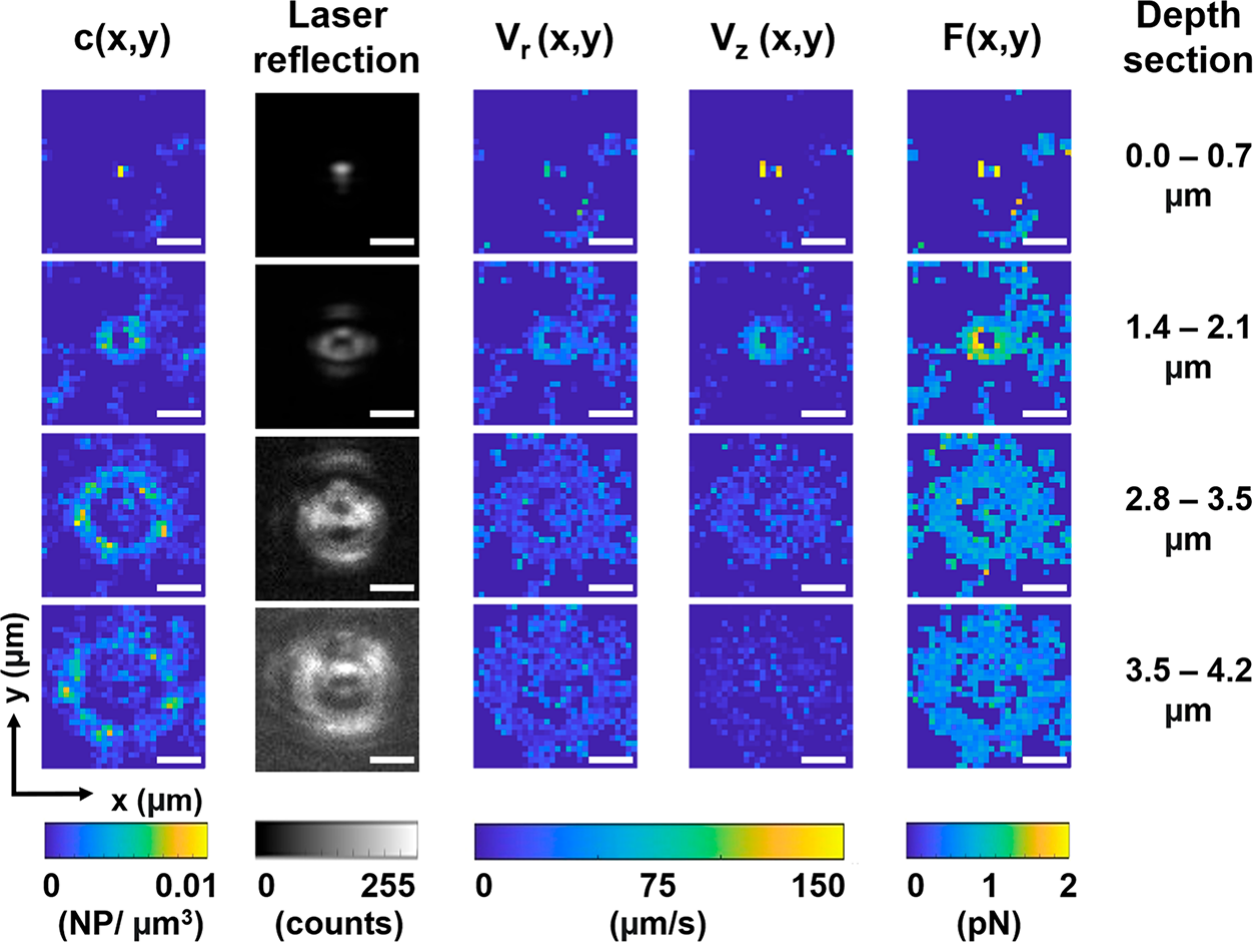
Images of the average concentration field *c*(*x*,*y*), 1064 nm laser reflection intensity, radial flow speed *v*_r_(*x*,*y*), axial flow speed *v*_*z*_(*x*,*y*), and optical force *F*(*x*,*y*) for six different depth sections. The length of the scale bar is 2 μm. The optical conditions are the following: laser power after the objective, 36 mW; NA, 1.20 (60× water immersion objective); circularly polarized laser.

From the NPs traces (Figure 2a), we can reconstruct the probability density function (PDF) by binning the data in 3D, which gives us an idea of what are the most likely position via which particles went through. This information can be then turned into maps of the concentration of NPs *c*(*x*,*y*), of radial speed *v*_r_(*x*,*y*), of axial speed *v*_*z*_(*x*,*y*), and of forces *F*(*x*,*y*). These different maps are shown in Figure 4. Without application of the laser field, the NPs present a random distribution [corresponding to constant *c*(*x*,*y*) or equal probabilities at every position]. However, when we switched on the trapping laser (nonequilibrium condition), the *c*(*x*,*y*) clearly showed an annular ring pattern, in which the NP concentration was much larger than before switching on the trapping laser. Moreover, the annular ring reveals the inhomogeneous spatial structure of the trapping laser beam inside the irradiated area.

To further rationalize the observed *c*(*x*,*y*) profiles, we compared them with the images obtained from the back reflection of the trapping laser at the interface for the different axial sections studied (Figure 4). Indeed, there is a clear resemblance between the NP concentration and the back reflection field (used as a proxy for the laser intensity). A high correlation between the light intensity and the NP concentration should be expected. Yet, the comparison with the back reflection image is not ideal because (i) the trapping laser intensity distribution is convoluted with the point spread function of the microscope and (ii) it is assumed that the trapping laser beam after reflection at the interface retains its symmetry. However, the theoretical laser profiles shown in Figure S3 also revealed a similar laser power distribution with several annular rings because of the interference of the focusing beam.

A closer inspection of the *c*(*x*,*y*) distribution reveals the presence of two concentric rings for the axial sections far away from the focus, thereby supporting the presence of two incorporation cones. The *c*(*x*,*y*) for the external cone is much larger than that of the internal one. Moreover, the internal cone collapses to a single point around 2.0–2.5 μm below the focus, which causes the aforementioned metastable trapping position (Figure 2b). This was also observed in the theoretical calculation of the laser profile where an internal annular ring converges to a single point at around 2.0 μm below the interface (Figure S3).

If the speed vector is decomposed into its axial (*v*_*z*_) and radial (*v*_r_) components (Figure 4), we observe that while the *v*_r_ hardly increases for the different *z* sections, the *v*_*z*_ is much faster (up to 5-fold) near the focus than farther away from it. The fact that *v*_r_ does not change significantly for different axial positions contradicts our intuition that the NPs should accelerate toward the center because of lateral gradient of the laser field. However, Figure S3 shows the simulated intensity laser pattern for different axial positions, where the electric field intensity is composed by several annular rings that periodically expand and contract. This structure is typically for tightly focused lasers and is referred to as a periodically converging pattern. Thus, the direction of the lateral optical force periodically changes, too. Therefore, *v*_r_ is more affected by the force fluctuation than *v*_*z*_, which hampers the NPs’ radial acceleration near the focus.

Figure 4 also shows the total optical force, estimated from eq 3, for the different axial planes. The optical force becomes larger near the focal spot where the photon density is largest. Near the focus, the axial component of the optical force is larger than its radial component. Inside the focal spot, an optical force of around 2 pN is estimated, which is in line with previous optical force reports calculated using other methods, such as trapping stiffness measurements under similar optical conditions.^44^ The force distribution also follows the distribution of laser power, thereby demonstrating again the importance of the laser profile in the optical potential field.

### 2.2 Controlling the Flow of Nanoparticles

In the previous section, we have demonstrated the potential of multiplane widefield imaging for studying the incorporation of NPs to a trapping side under one specific optical condition. We can now use the same system and method to study the effect of different optical conditions on the 3D incorporation dynamics.

#### 2.2.1 Laser Polarization Leads to Different Nanoparticle Distributions

The first evaluated optical condition is the laser polarization, which affects the intensity and phase profile distribution for tightly focused beams.^45^ Previously, Chiu et al. took advantage of complex laser polarization combinations to enhance the induced optical force, and Kudo et al. studied the effect of laser polarization on the formation of optically induced assemblies.^19,46^ However, to the best of our knowledge, no one has directly visualized its effect during the optical trapping process.

**Figure 5:**
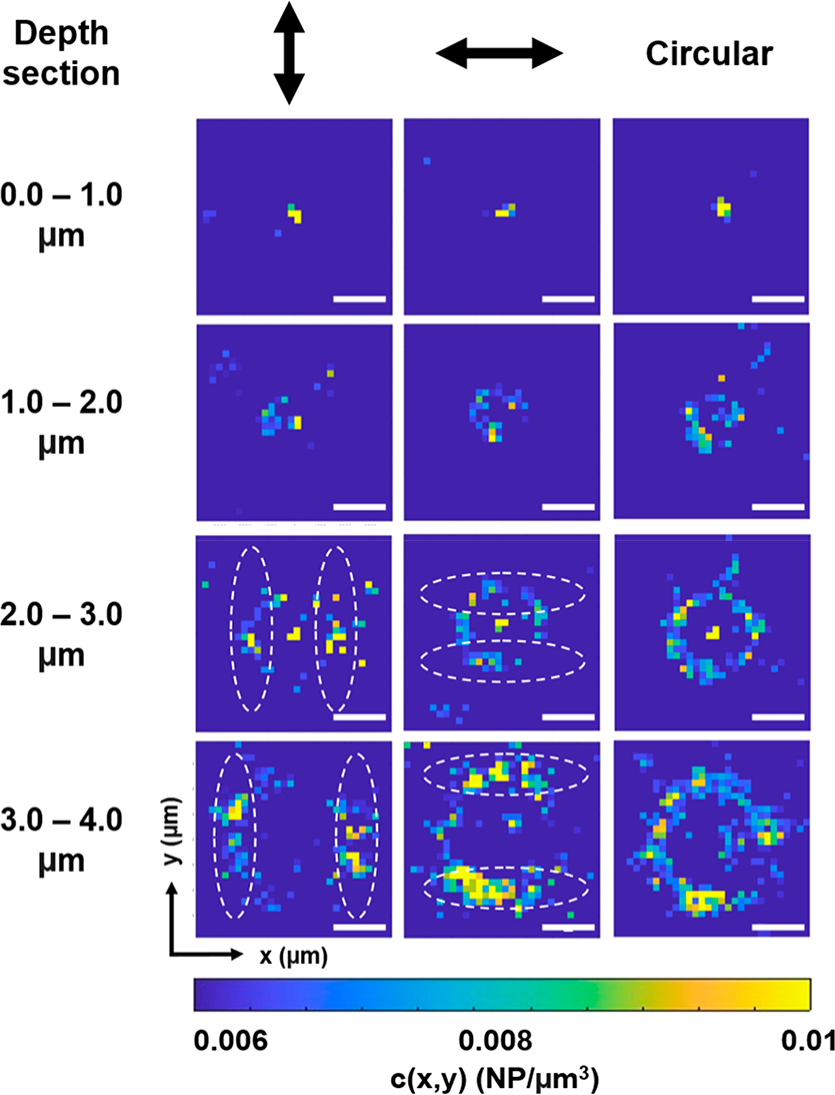
Average concentration field *c*(*x*,*y*) for three different laser polarizations (linear horizontal, lineal vertical, and circular) for different depth sections. The length of the scale bar is 2 μm. As a visual aid, we have saturated the color scale to show only high-concentration regions and included dashed lines at the regions where the *c*(*x*,*y*) field is larger (see Figure S5 for unsaturated color scale from 0 to 0.01 NP/μm^3^).

In Figure 5, we compare the incorporation phenomena using linearly and circularly polarized trapping laser light. Of note, with the aim of helping the reader to notice the differences, the color has been scaled from 0.006 to 0.01 NP/μm^3^ (see Figure S5 for the original color scale from 0 to 0.01 NP/μm^3^). As previously mentioned, circularly polarized light yields an isotropic power distribution inside the tightly focused annular rings, and therefore, no preferential incorporation regions are observed in those rings. Conversely, linearly polarized light yields an anisotropic distribution of NP positions. The preferential motion paths are oriented perpendicular to the direction of the laser light polarization. This experimental observation is also supported by the laser profile theoretical calculations (Figure S6), in which the calculated laser power within the rings is more intense in regions perpendicular to the laser light polarization. The incorporation speed also follows the same distribution pattern, and we observed the largest flow speed in the regions perpendicular to linear laser polarization (Figure S7). Moreover, if we rotate the linear laser polarization by 90 degrees (i.e., from horizontal to vertical), we observe that the preferential incorporation area also rotates by 90 degrees. This preferential incoming area is clearer away from the focus, where the linear polarization has a larger impact on the power distribution for tightly focused beams, as shown in our calculations in Figure S6.

#### 2.2.2 Objective Lens Numerical Aperture Changes the Incorporation Angle

The numerical aperture (NA) strongly influences the laser beam profile in 3D. On the one hand, lasers focused by a low NA objective can be correctly described by a simple Gaussian mathematical model, while lasers focused by a larger NA need more complex models, such as the angular spectrum representation.^47,48^ On the other hand, the NA changes the divergence angle of the laser beam. Therefore, study of the influence of the NA is crucial to understanding the impact of the beam shape on optical trapping incorporation.

A simple approach to change the effective NA is to shrink the laser beam through an iris diaphragm before it enters the back aperture of the objective, hence effectively not fully filling the back aperture of the objective. The diameter of the objective back aperture is 7 mm. Herein, we compared three different conditions: the diaphragm fully open (ray-optical model) and partially closed with beam diameters of 4.5 and 3.0 mm. It is worth mentioning that no stable trapping spot was achieved for openings smaller than 3.0 mm, which is consistent with the lower trapping capabilities of laser beams focused by low NA lenses. Note that the trapping laser power after the objective was fixed to 60 mW to enable the comparison between the different optical conditions.

**Figure 6:**
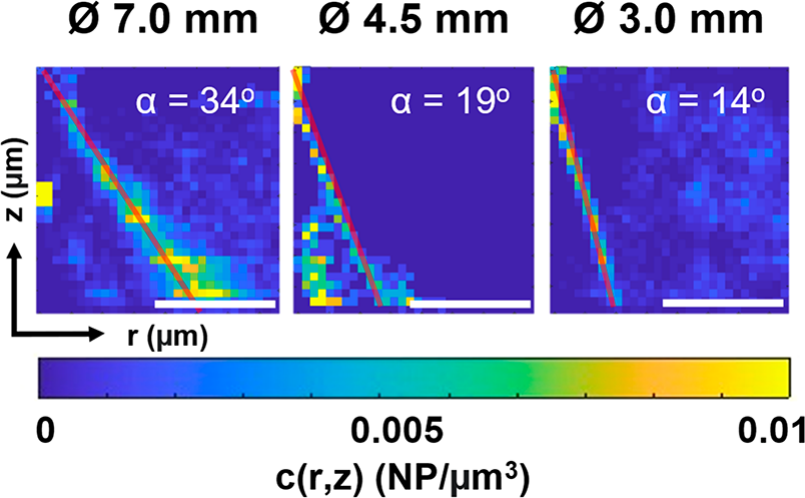
Average concentration field *c*(*r*,*z*) for different effective NA conditions. The effective NA is modified by changing the trapping laser beam size through an iris diaphragm. The length of the scale bar is 2 μm.

Since the incorporation angle is the main factor here, the data is presented as *c*(*r*,*z*) which is the *z* dependence of the radial average of the concentration field. Figure 6 shows that varying the effective NA leads to significant modifications in the NP *c*(*r*,*z*) along axial and radial coordinates. We found that the incorporation angle for the external annular ring is around 34°, 19°, and 14° for beam diameter of 7.0, 4.5, and 3.0 mm, respectively. This decrease is expected because the effective NA becomes smaller upon closing the iris diaphragm. It is worth mentioning that the theoretical focusing angle for a fully open diaphragm is around 64°, according to the NA of the objective used. This calculated angle is almost two times larger than the incorporating angle we observed, and therefore, the observed incorporation channel angle cannot directly be explained by the objective’s NA value because it expresses the focusing angle of a lens at the geometrical optics level of theory; however, near the laser focus, the focusing angle becomes almost parallel to the optical axis because of diffraction limitations.

Thus, to better understand the role of the NA, we calculated the electric field of the trapping laser for the three tested optical conditions (Figure S8). The electric field intensity was normalized for each *z* plane to visualize how the relative position of the local maxima changes along the radial and axial position. Unlike the periodically converging pattern observed for the fully open condition, a simple Gaussian-like distribution was observed for the 3.0 mm pinhole condition. The experimentally observed incorporation angle was similar to the angle described by the contour defined by 20% of the simulated electric field intensity for each optical condition (Table S1). Thus, the incorporation channel has a relatively constant gradient/scattering force ratio. It should be noted that when the pinhole is open (i.e., large NA), the incorporation channel, especially away from the focus, is broad because of the multiple expansion and contraction cycles of the different annular rings attributed to the periodically converging laser pattern. Conversely, under an optical condition, where the periodically converging laser pattern is not present (i.e., 3.0 mm pinhole opening), a sharp incorporation channel is observed, which confirms the importance of the laser pattern on the shape of the channel.

Figure S9 shows the spatial distribution of axial speed (*v*_*z*_). First, we observed that the *v*_*z*_ distribution was much more homogeneous for a small effective NA, which is in agreement with the calculated angular spectrum (Figure S8). Second, larger *v*_*z*_ speeds were achieved when far outside the trapping laser focus at a small effective NA. These two observations can be explained by the fact that the area of the cone far from the focus is smaller for a low effective NA. Consequently, the photon density (for the same laser power) will be larger, thereby resulting in a stronger induced optical force, which implies a larger *v*_*z*_. Moreover, the area differences between different axial sections will be smaller for a low effective NA, thereby leading to a sharper *v*_*z*_ distribution. The experimental findings show how sensitive the incorporation pathways are with respect to the laser beam profile, and therefore, it is essential to know the 3D laser profile with the highest accuracy for fully understanding the optical trapping properties.

Finally, additional experiments are currently being conducted in which the incorporation angle is reduced to less than 8° when the viscosity of the medium increases 4-fold by addition of 40% of glycerol (Figure S10). This preliminary experiment indicates that physicochemical properties of the environment also affect the incorporation angle, thereby requiring a comprehensive study to fully understand the phenomenon.

#### 2.2.3 Laser Power Leads to Sharper Channels

The most direct way to increase the trapping intensity is to increase the laser power. Figure S11 shows the average concentration field *c*(*r*,*z*) of particles for different trapping powers (36, 60, 120, and 240 mW after the objective). A visual inspection of the *c*(*r*,*z*) reveals that the shallow cone width is sharper for larger trapping laser powers, which indicates that a spatially more confined channel is formed inside the irradiated cone (Figure S11). This is likely because of the observed increase in speeds *v*_r_ and *v*_*z*_, as shown in Figure S12. Indeed, as the laser power is increased, the directional motion of NP driven by the induced optical force becomes more significant compared with Brownian displacements, thereby yielding straighter incorporation trajectories. We note here that the spatial distribution and the annular ring formation are not affected. These results could be expected, as increasing the trapping laser power should only increase the intensity of the optical field but not change its shape.

A particularly relevant quantity here is the ratio between the optically driven velocity (*v*) and the diffusion velocity (*D*), given by the Péclet number (*Pe*; eq 2):
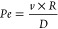
2

Figure S13 shows the local *Pe* number map distribution (*r*,*z*) for the different trapping laser powers tested. As the laser power is increased, the *Pe* reaches about 100, which clearly indicates that the optical driving dominates the NP motion there. Far from the focus, Brownian motion dominates, and *Pe* decreases below 1. It is interesting to further analyze the optical *Pe*. Use of the Stokes–Einstein relation for the particle diffusion coefficient (eq 3) and the drag force (eq 1), the *Pe* number can also be expressed as eq 4.
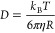
3
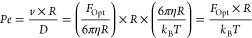
4

The *Pe* is just the ratio between the work delivered by the optical force in moving one particle along its own radius (*F* × *R*) and the thermal energy (*k*_B_*T*). An interesting conclusion of this relation is that the *Pe* is independent of the solvent viscosity η under this condition because the viscosity is canceled by its appearance in both the numerator and the denominator of the equation. Increase of the laser power permits us to analyze several relevant quantities, such as the work done by the laser field per particle and the relevance of collective hydrodynamic interactions between NPs. From a thermodynamic perspective, the potential energy of particles can be related to their spatial distribution via Boltzmann statistics. Thus, the trapping laser “free energy” per particle can be estimated from the local concentration field *c*(*r*), which is particularly relevant for the trapping paths. The small NP concentration permits us to assume a high dilution condition for the NPs, in the sense that particle–particle interactions (collisions) can be completely neglected while NPs still can interact through the fluid (i.e., hydrodynamics). In this case, the effective free energy field manifests as eq 5, where negative *U*_trap_(*r*) values mean attraction. Of note, *U*_trap_(*r*) is an equilibrium property, which does not depend on hydrodynamics and, as expected, only depends on the local NP concentration.
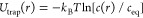
5

**Figure 7:**
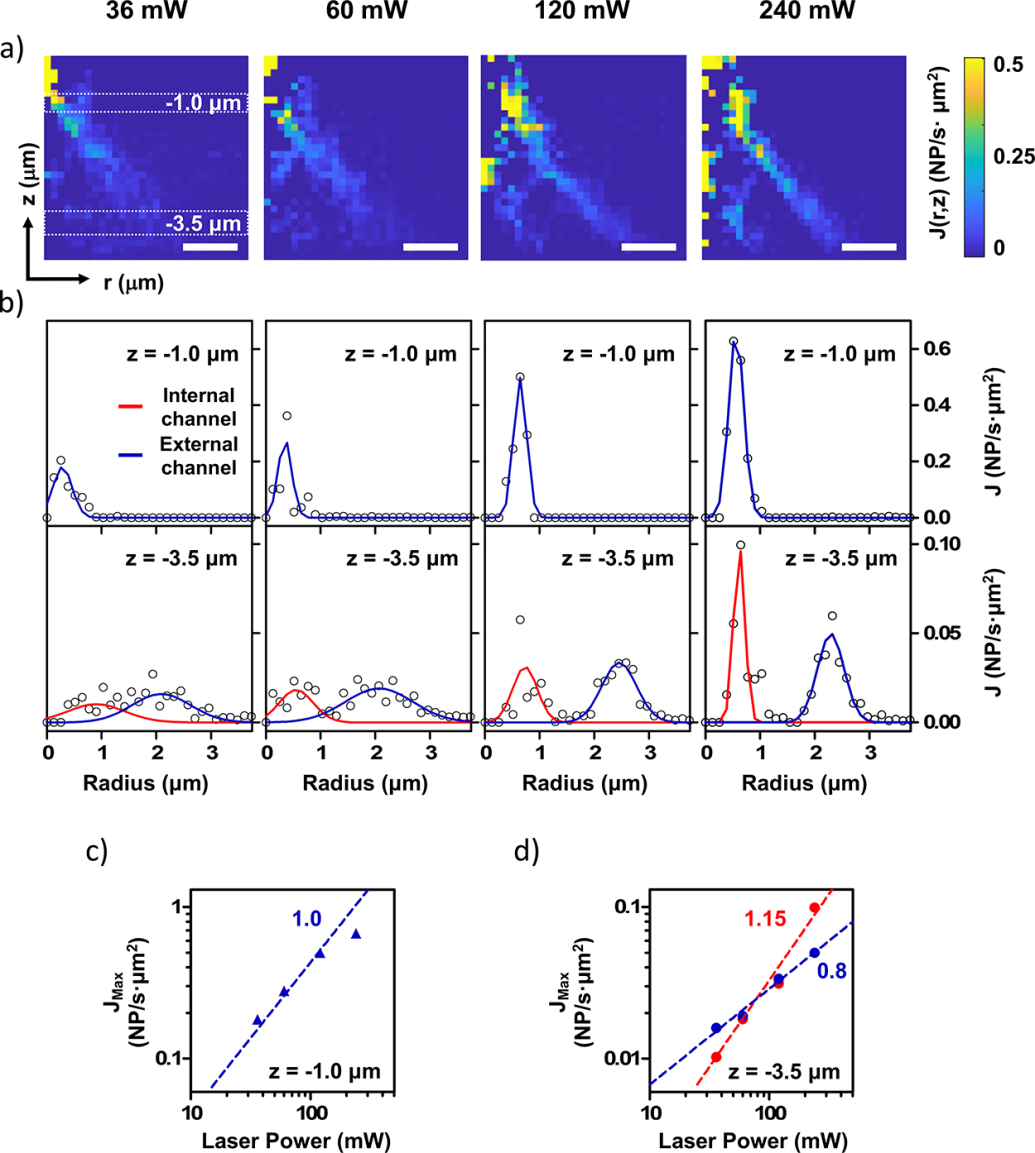
NP flux analysis for different trapping laser powers (36, 60, 120, and 240 mW). (a) NP flux map *J*(*r*,*z*). (b) NP flux variation along the radial coordinate for two different depth sections: top (*z* = −1.0 μm), bottom (*z* = −3.5 μm). Both internal (blue) and external (red) NP channels are fitted using a Gaussian function. (c,d) Maximum flux dependence with the trapping laser power for both internal (red) and external (blue) NPs channels at a depths of −1.0 and −3.5 μm (c and d, respectively). Numbers beside the dashed lines indicate the exponents of the best fit to power laws for the inner channel (red) and outer channel (blue). The length of the scale bar is 1 μm.

For the largest laser power used, the NP concentration in the trapping paths near the focal spot is between 10 and 50 times larger than the equilibrium concentration *c*_eq_ = 5 × 10^9^ NPs/mL, which corresponds to trapping energies (*U*_trap_) between −2.3 and −4.0 *k*_B_*T* per particle. These energy values are large enough to overcome the thermal fluctuation and induce the preferential channels before reaching the trapping site. It is worth mentioning that the *U*_trap_ is just the conservative part of the laser’s work, which describes the force that traps the NPs in the optical paths. The other significant contribution to the laser work, which is the responsible for creating the NP currents, is not conservative and, therefore, it cannot be described by an effective optical free energy; however, it is related to the Poynting vector.^36,42^ Notably, the observed large *Pe* indicates that this form of the laser energy is significant inside the paths. To understand this second advective contribution due to the photoinduced current flows, we evaluated the NP local flux [**j**(**r**); eq 6]
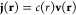
6

where *c*(*r*) is the NP concentration field and **v**(**r**) is the NP velocity field (average velocity at each pixel; Figure 7a). Note that boldface letters indicate a vector quantity. The NP flux is about 10 times larger in the outer cone, and the maximum flux is located close to the focal spot. Figure 7c,d plots the NP flux averaged over the optical path width where the NP concentration presents a Gaussian peak (see Figure 7b) against the laser power (*P*). In absence of hydrodynamic interactions, *J* should linearly increase with the laser power. However, far from the laser focus, we observe a mild superlinear scaling of the flux, *J ∼ P*^1.15^, which confirms the presence of hydrodynamic interactions. Note that the local NP volume fraction in the paths is about ϕ_path_ ≈ 2 × 10^–4^, which is slightly above the threshold for the predicted collective motion;^32^ hence, collective hydrodynamic effects are mild but expected. The particles get trapped at the focus. Close to the focus we observe a sublinear scaling of the flux with *P*, quite probably because of this effect (Figure 7c,d). It must be mentioned that for *P* = 240 mW, the time required for a NP to move from the maximum flux location to the focal spot is similar to the experimental time to take a snapshot (5 ms). Therefore, close to the spot, the NP velocity might be somewhat underestimated.

### 2.3 Relevance of 3D Microscopy to Study the Dynamics of Nanoparticles Inside an Optical Field and Its Future Perspectives

Optical trapping is an intrinsically three-dimensional phenomenon. In most optical systems, the optical tweezers unit is coupled with an inverted microscope, and therefore, the propagation of the trapping laser beam mainly occurs in the axial direction (*z*). However, these microscopes typically record a single (*x*–*y*) plane image at a time where the axial information is contained in the shape of the point spread function of the emitters. Such encoded information has been used by several groups to qualitatively estimate the axial position of trapped objects (e.g., defocusing extent of the emitter particle, point spread function engineering, holographic images, or the particle contrast on transmission images).^49−52^ However, these strategies normally lack either spatial accuracy, axial depth range, or are limited to low concentrations of particles. An alternative approach is the use of a dual objective microscope, where one objective is used for optical trapping and the other for imaging purposes.^53,54^ However, this approach fails for single particle tracking in highly dynamic systems because of the dead time issues during the *z* stack acquisition, which precludes obtaining reliable volumetric images with proper temporal resolution.

We overcame these limitations using our multiplane widefield microscopy combined with an optical trapping unit. In a former work, we estimated the single-particle tracking (SPT) precision of our multiplane imaging microscope to be 10–15 nm for *x*–*y* and 27 nm for *z*.^39^ These precisions are high enough to obtain reliable 3D particle incorporation traces and to estimate their incoming speed. Moreover, the NPs can be tracked for a longer time in comparison with conventional single-plane widefield microscopy since they do not easily go out of focus. Thus, the presented axial information that is not readily obtained using conventional imaging systems is often disregarded. Indeed, having clear images of what happens simultaneously at different depths revealed completely unreported phenomena such as the observation of 3D hydrodynamics incorporation channels, which can be directly linked to theoretical approaches in light–matter interaction. Although another approach has also been proposed to study the 3D motion of MPs inside optical guiding beams by coupling a right-angle microreflector to obtain the axial image,^55^ this technique is much more complex in terms of optical elements and lacks the required brightness, accuracy, magnification, and time resolution for obtaining super-resolved 3D traces of fluorescent nanoparticles at and nearby the trapping laser focus.

Our experimental setup can decompose the 3D velocity of each NP into its corresponding axial and radial components. The Stokes drag relation immediately provides access to the optical driving force acting on each NP. From an ensemble of independent measurements, we could reconstruct the average concentration [*c*(*x*,*y*,*z*)], the axial and the radial velocities [*v*_r_(*x*,*y*,*z*) and *v*_*z*_(*x*,*y*,*z*), respectively], and the force [*F*(*x*,*y*,*z*)] fields. Indeed, the rate of work done by the laser beam in moving the NPs can be directly evaluated from the product *F*_Opt_ × *v*. Further validation of these measured fields with theory to develop/refine the current models is essential to fully understand the opto-hydrodynamics of the system.^36^ For instance, in the present experiments, we observed that the flux of NPs toward the focal spot slightly deviates from a linear relation with the laser power, which suggests a mild effect of hydrodynamic coupling among the NPs because of the propagation of optical forces to the solvent flow. At larger NP concentrations, these collective hydrodynamic effects are intertwined with the secondary optical forces (scattering), which leads to quite complex dynamics. It is interesting to note the formation of two preferential channels along which NPs move that directly reflect the nonhomogeneous structure of the optical field.^42^

We foresee that our 3D imaging technique will also be useful for unraveling the relation between the laser beam structure, the induced particle dynamics, and the opto-hydrodynamics of the system by resolving the 3D dynamics of the particle outside complex optical potentials. Indeed, the application of widefield multiplane imaging is not only limited to the phenomena that occur inside the irradiated area, but it can also be used to study how an optical field can induce other forces (e.g., Marangoni and convection forces) as a result of the alteration of the properties outside the irradiated area (e.g., temperature, flow). Moreover, the use of multiplane widefield microscopy can be further expanded to study the 3D motion of particles under other force potentials (e.g., electric, magnetic, gravitational), thereby endowing a huge potential to the reported technique.

## 3 Conclusions

We have demonstrated the potential of multiplane widefield microscopy to study dynamical phenomena inside an optical trapping field. We have directly imaged the 3D incoming trajectory of NPs toward a stable optical trapping spot. Indeed, we can observe and describe the NPs’ incoming trajectory as following a shallow cone distribution with two axial annular rings, which can be qualitatively linked to the power distribution in a tightly focused laser beam. Further information can be obtained by detailed analysis of the position of each particle at different imaging times. As an example, the 3D speed (and its monodirectional components) has been calculated, which has revealed that the axial/radial speed ratio depends on the depth with respect to the focus, and has provided direct access to the optical force field. The 3D analysis has been further expanded to other optical experimental conditions (e.g., laser power and polarization, effective numerical aperture), thereby confirming the importance of the 3D tightly focused laser power distribution on the particle incorporation phenomenon. However, other observed phenomena, such as the individual particle incorporation through some nonlineal preferential channels, cannot be explained only by the 3D laser power distribution, which advocates that the hydrodynamic interactions modify the incorporation trajectory. Three-dimensional imaging permits describing incorporation collective dynamics, thereby facilitating a more thorough comprehension of the intertwined optical and hydrodynamic interactions of dielectric particles inside a tightly focused laser beam.

## 4 Materials and Methods

### 4.1 Sample Preparation

We used commercially available fluorescent spherical polystyrene (PS) NPs with a diameter of 200 nm (FluoSphere, 505/515, carboxylate-modified, ThermoFisher Scientific). The sample was prepared by sandwiching 8 μL of a 1000× dilution of the commercial NP suspension between two clean coverslips with a 120 μm depth spacer (Grace Bio-Laboratories). The coverslips were cleaned using ozone treatment for 60 min. To image individual particle incorporation events, the equilibrium NP concentration was set to a value of *c*_eq_ = 5 × 10^9^ particles/mL, which corresponds to a particle average volume fraction of ϕ = 2.1 × 10^–5^.

### 4.2 Combination of a Widefield Multiplane Microscope with an Optical Tweezer

Multiplane imaging is based on the fact that the sample plane in focus at the detector depends on the distance between the tube lens and the detector. In other words, the depth of the imaging plane is controlled by changing the optical path length. Therefore, a 3D image can be recorded if the imaging beam is split into multiple beams and the resulting images are acquired simultaneously. Several strategies have been used to split the imaging beam, from the simplest approach using a beamspliter^56^ to more complex optics, such as the use of prism or diffractive networks.^57−60^ In a previous technical note, we described the use of a widefield multiplane microscope (Figure 1) optimized for fast acquisition rates inspired by the work of Geissbuehler et al.^58^

Briefly, a 488 nm laser line was used for widefield illumination by focusing the laser beam at the back focal plane of the objective lens. The fluorescence emission was collected by a water immersion objective lens (NA 1.20, 60×, Olympus UPlanSApo60XW) and filtered with a band-pass filter (ZET488/561m, Chroma Technology) and a 1010 nm short-pass optical filter (FF01-1010/SP-25, Semrock) to remove the excitation and the trapping laser back reflection components, respectively. The emitted light went through a set of lenses in telecentric 4f configuration [objective (*f* = 3.3 mm) – lens 1 (L1, Thorlabs, plano-convex, *f* = 140 mm) – field stop – lens 2 (L2, Thorlabs, plano-convex, *f* = 140 mm) – tube lens (TL, Thorlabs, plano-convex, *f* = 200 mm)]. The field stop was used to control the size of the image. Between the tube lens and the cameras, we placed a proprietary prism, which splits the entering photon flux into eight different beams with slightly different optical path lengths. The distance between consecutive planes was fixed because of the prism geometry and was roughly 580 nm, which yielded an axial range of approximately 4 μm. Moreover, through the utilization of a high-frequency NBS resolution test target (cycle size from 1.0 mm to 4.4 μm), it was confirmed that the magnification remained equal across all planes. The different imaging planes were recorded by 2 scientific complementary metal–oxide–semiconductor (sCMOS) cameras (4 imaging planes for each camera, Orca Flash 4.0, Hamamatsu Photonics Inc.). To ensure fast acquisition rates (200 fps) and correct synchronization of the two cameras, we triggered the acquisition via a National Instruments board (NI, USB-6343) controlled by home-built software written in Labview. The imaging volume was 500 pixels × 500 pixels × 8 planes (50 × 50 × 4 μm^3^).

To determine the exact axial position of the 8 imaging planes, we used a sample of 0.2 μm diameter fluorescent beads (FluoSphere, 505/515, carboxylate-modified, ThermoFisher Scientific) spin-casted onto a glass coverslip. The concentration was sufficient to image 50–100 beads within a single field of view while still allowing for clear individual separation. The sample was then scanned along the axial direction, spanning the entire imaging volume, and the image gradient was extracted for each plane as a function of the axial stage position (as depicted in Figure S14). Since the image gradient was maximized when the focus was sharpest, we were able to accurately determine the position of the planes by fitting the *z* dependence of the image gradient.^39^ Of note, we covered the spin-casted fluorescent beads with a hydrogel (>99.9% water) to avoid a longitudinal magnification aberration due to a refractive index mismatching with the sample.

The laser trapping system was installed on the same inverted multiplane widefield microscope (Figure 1). A 1064 nm continuous wave laser was guided to the sample and collimated via a beam expander that ensured the filling of the back aperture of the objective. Then, the trapping laser was focused inside the NP suspension by the water immersion objective lens. The laser was focused deeply enough inside the solution to avoid surface effects. Optical trapping conditions were controlled as follows: (i) the laser power after the objective lens was controlled using a half-wavelength plate combined with a polarizing beam splitter; (ii) the laser polarization was controlled using a half or a quarter-wavelength plate for linear or circular laser polarization output, respectively; and (iii) the effective objective numerical aperture (NA) was changed by reducing the size of the trapping laser beam using a diaphragm before the back aperture of the objective and, hence, controlling the degree of filling of the aperture.

The image processing and the single-particle tracking (SPT) of the fluorescent particles were performed using a 3D phasor analysis, as previously reported.^36,61^ The software is freely available from https://github.com/CamachoDejay/polymer3D.

### 4.3 Calculation of Tightly Focused Laser Beam Profiles

To compare our experimental results with theory, we simulated the tightly focused beam profile for the different optical conditions employed using the angular spectrum representation. This representation is a rigorous and powerful method to describe the laser beam propagation and light focusing.^62^ The incident field was decomposed into plane waves and Fourier-transformed into *k*-space to obtain their angular spectrum. Then, we calculated the focused laser field by integrating these plane waves to obtain their superposition after propagation within the boundary conditions determined by the optical setup, such as the NA value. The propagation of waves could be easily expressed in the Fourier space, and the interferences between the waves were considered when they were integrated, thereby yielding an accurate description of the focused field.

The incident field (E_inc_) was refracted by a reference sphere with a radius equal to the focal length, representing a lens which focuses the light to the focal spot. The refracted far-field (E_∞_) after the sphere was formulated in eq 7.
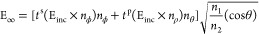
7where *t*^s^ and *t*^p^ are the Fresnel transmission coefficients for the refraction of s- and p-polarized light, respectively; *n*_ϕ_ and *n*_ρ_ are the unit vectors for describing s- and p-polarized field before refraction, while *n*_ϕ_ and *n*_θ_ are those after refraction; *n*_1_ and *n*_2_ are the refractive index before and after the reference sphere, respectively; and θ is the focusing angle (see Figure S15).

The field (E) near the focus was obtained by integrating all these *k*-components after their propagation to the focus. The resulting field is described with a cylindrical coordinate system (eq 8).
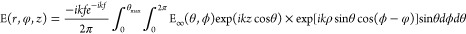
8where *k*, *f*, and *z* are the wave vector, focal length and the displacement between the calculated plane and the focus, respectively; *r* and φ are the radius and the angle for the target position with cylindrical coordinate notation, while θ and ϕ are the angles for the light propagation; and θ_max_ refers to the largest focusing angle, namely the NA value. The geometrical scheme of this calculation model is found in Figure S15.

## Supporting Information Available

Additional supplementary figures: the geometrical scheme of the calculation model and the definition of its coordinates for the laser beam profile simulation; simulated laser beam profiles, average concentration, and average speed maps for different optical conditions; detailed description of the multiplane widefield microscope; representative traces for the incorporation of fluorescent polystyrene NPs; and supplementary bibliography (PDF)
Motion of particles up to a depth of 4 μm below the trapping site on the first imaging plane (AVI)

## Supplementary Material

nn2c11753_si_001.pdf

nn2c11753_si_002.avi
