# Supplementary material for: Unravelling 3D Dynamics and Hydrodynamics during Incorporation of Dielectric Particles to an Optical Trapping Site
Source: ACS Nano. 2023 Feb 17;17(4):3797–808. doi: 10.1021/acsnano.2c11753 (PMC10623636; doi:10.1021/acsnano.2c11753)
Supplement: Supplementary file 1 — nn2c11753_si_001.pdf [file nn2c11753_si_001.pdf]

Supplementary information

Unravelling the 3D dynamics and hydrodynamics during incorporation of dielectric particles to an optical trapping site.

Boris Louis,[1,3,+] Chih-Hao Huang,[2,+] Rafael Camacho,[3] Ivan G. Scheblykin,[4] Teruki Sugiyama,[2,5*] Tetsuhiro Kudo,[2] Marc Melendez[6], Rafael Delgado-Buscalioni[6], Hiroshi Masuhara[2,7,*] Johan Hofkens,[1,8,*] Roger Bresoli-Obach[1,9,*]

[+] These two authors contributed the same

* Corresponding authors: Teruki Sugiyama (sugiyama@nycu.edu.tw); Hiroshi Masuhara (masuhara@masuhara.jp); Johan Hofkens (johan.hofkens@kuleuven.be); Roger Bresoli-Obach (roger.bresoli@iqs.url.edu)

[1] *Molecular Imaging and Photonics, Department of Chemistry, KU Leuven, Celestijnenlaan 200F, Leuven, 3001, Belgium.*

[2] *Department of Applied Chemistry, National Yang Ming Chiao Tung University, 1001 Ta Hsueh Road, Hsinchu, 30093, Taiwan.*

[3] *Center for Cellular Imaging, Core Facilities, the Sahlgrenska Academy, University of Gothenburg, Medicinaregatan 5A-7A, Box 413, Gothenburg, 40530, Sweden.*

[4] *Division of Chemical Physics and NanoLund, Lund University, Kemicentrum Naturvetarvägen 16, P.O. Box 124, Lund, 22100, Sweden.*

[5] *Division of Materials Science, Nara Institute of Science and Technology, 8916-5 Takayamacho, Ikoma, Nara, 630-0101, Japan.*

[6] *Departamento de Física Teórica de la Materia Condensada, Institut for Condensed Matter (IFIMAC), Universidad Autónoma de Madrid, Campus de Cantoblanco, Madrid, 28049, Spain.*

[7] *Center for Emergent Functional Matter Science, National Yang Ming Chiao Tung University, 1001 Ta Hsueh Road, Hsinchu 30093, Taiwan.*

[8] *Max Planck Institute for Polymer Research, Ackermannweg 10, Mainz 55128, Germany.*

[9] *AppLightChem, Institut Químic de Sarrià, Universitat Ramon Llull, Via Augusta 390, Barcelona, 08017, Catalunya, Spain.*

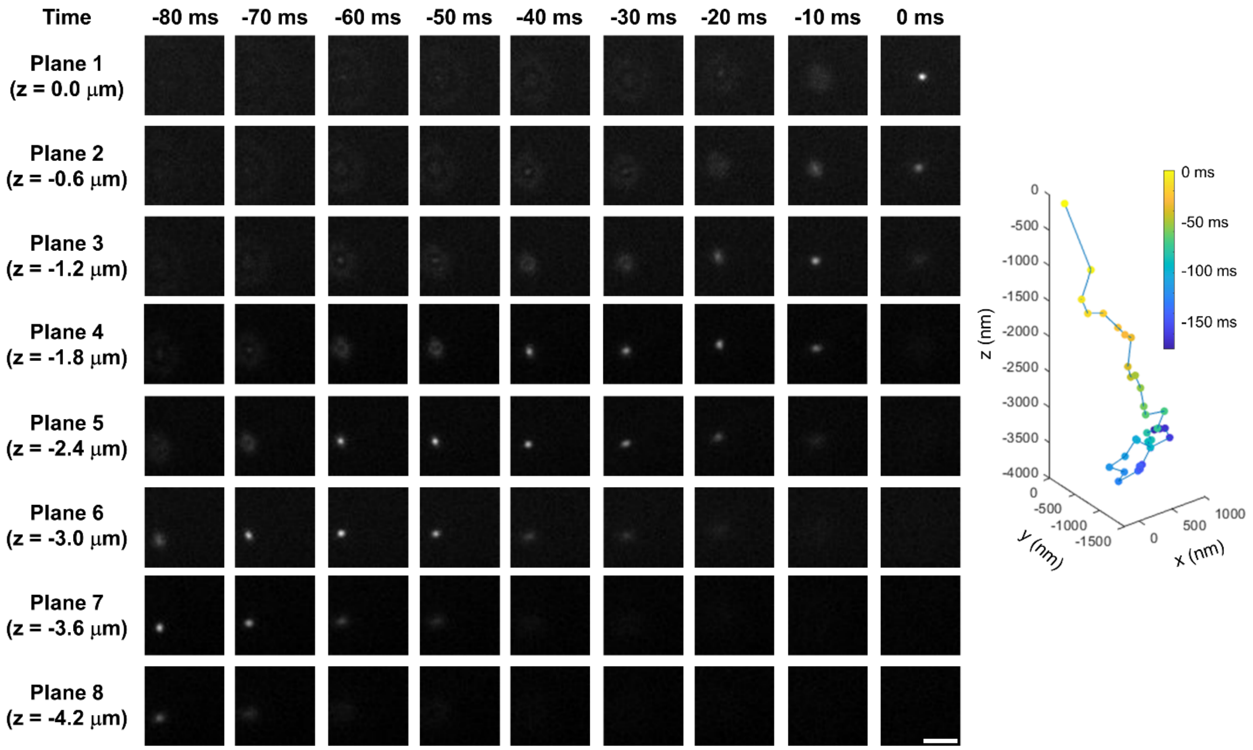

Figure S1 Raw data acquired for the incorporation of a single fluorescent polystyrene nanoparticle in eight different depth imaging planes. The white scale bar is 2 μm. In this figure, we printed the different image captures every 10 ms to properly resolve the motion and not to oversample the figure, although we acquired the data at a much faster frequency (200 Hz). The polystyrene nanoparticle is clearly observed during 3-4 frames for each selected time. The detection and localization of the particles are performed as described previously.[1] Briefly, the emitters were detected using the generalized-like hood ratio test developed by Sergé et al.[2] Once the particles were detected, they were accurately localized using a 3D phasor-based approach.[3] The x-y position of the particle is obtained by the first Fourier coefficient, which contains information about the modulus and the angle of the particle position. The x-y position is only calculated for the brightest imaging plane (i.e. the imaging plane where the emitter is focused). The z-position of the particle is obtained by fitting the dependence of the phasor magnitude across the different imaging planes with a 1D-Gaussian function. Our previous work validated this method with accuracies of 10-15 nm for x-/y- direction and 27 nm for z-direction under similar imaging conditions.[2] Particle tracking was performed by minimizing the sum of the displacements of all particles within a certain radius.[4] This was performed via the Munkres algorithm.[5] The 3D graph on the left shows the 3D trajectory after detecting, localizing, and tracking the same example particle.

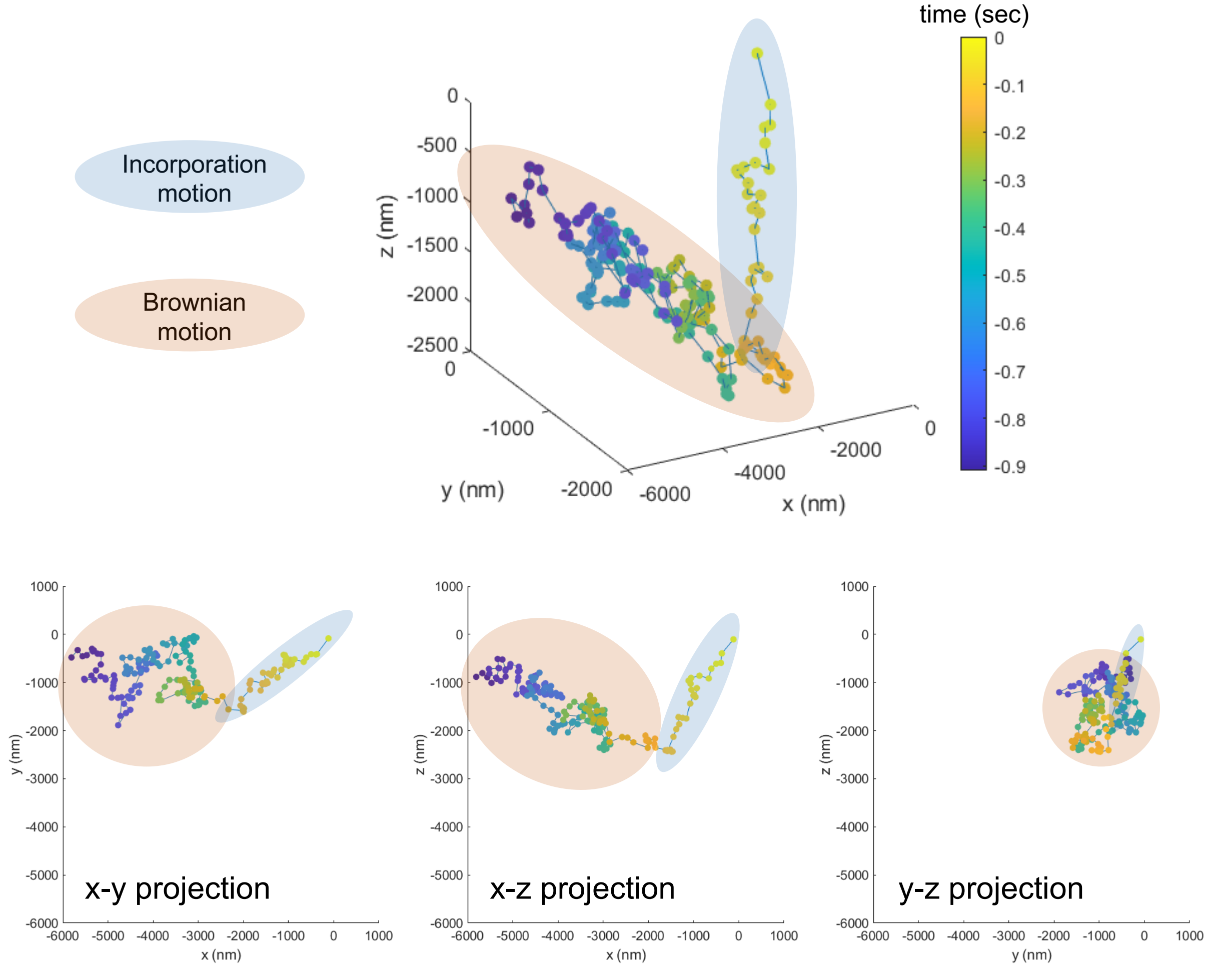

Figure S2 A representative example for the incorporation of a fluorescent polystyrene nanoparticle. Top: 3-dimensional representation. Bottom: 2-dimensional (x-y, x-z and y-z) projection. The color scale indicates the time before the nanoparticle reaches the focal spot. We can clearly observe that the nanoparticle trajectory follows two different motion stages. First, it moves randomly (Brownian motion) until it arrives inside the irradiation trapping cone. Once there, the nanoparticle goes to the focal spot following the external annular laser ring as explained in the main text. We filtered the incorporation traces from all the detected traces taking advantage of the fact that the incorporation phenomenon presents a high-directional motion towards the focal spot.

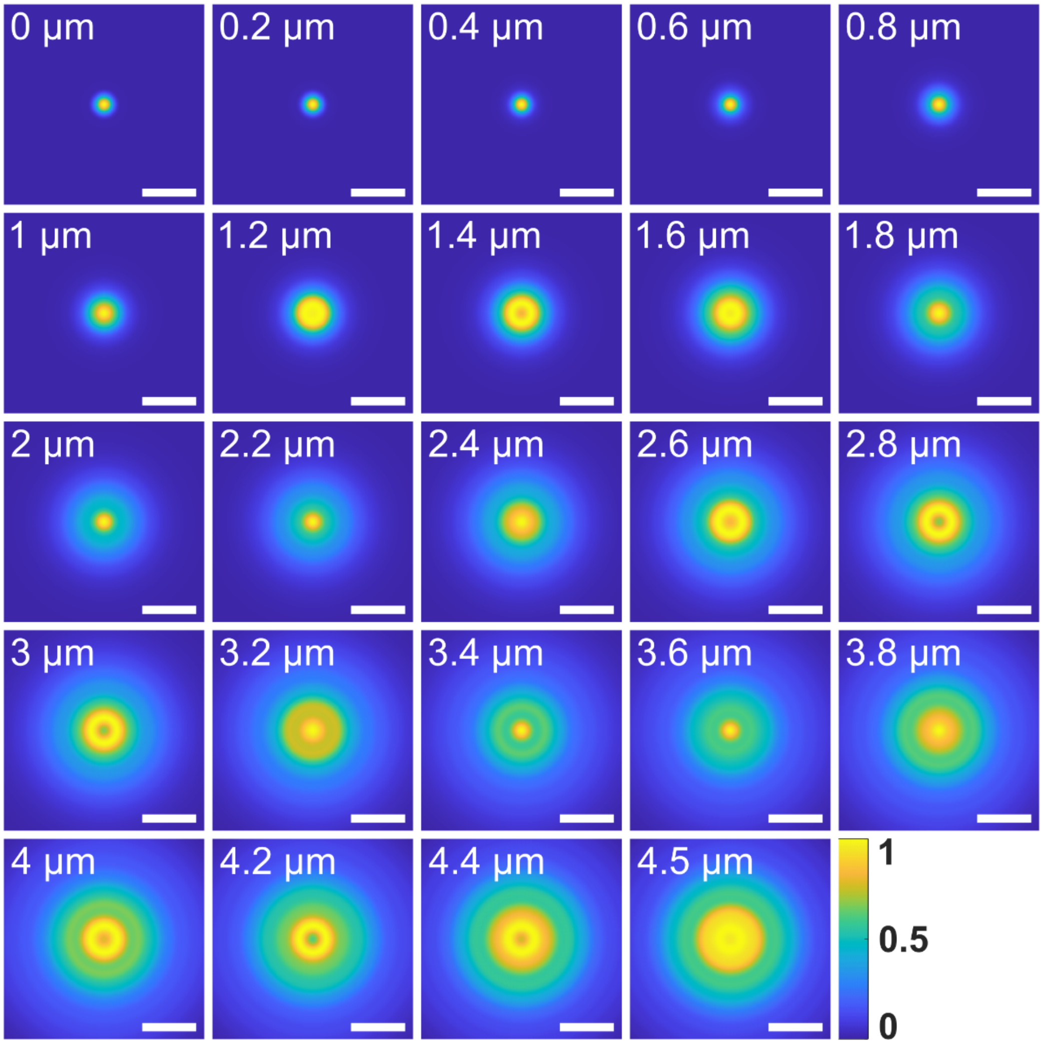

Figure S3 Calculated electric field intensity distribution for a tightly focused laser beam with circular polarization for different depth sections. The electric field intensity is normalized for each depth plane. The white scale bar corresponds to 2 μm. Of note, the direction of the circular polarization (clockwise or anticlockwise) does not affect the electric field distribution.

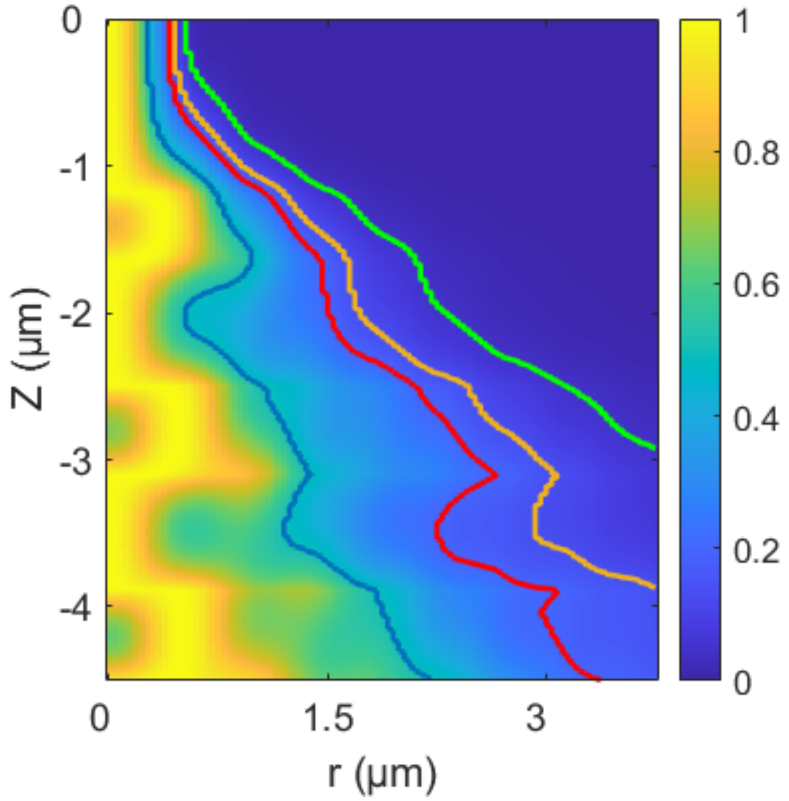

Figure S4 Side view of the calculated electric field intensity distribution for a tightly focused laser beam with circular polarization. The intensity is normalized at each depth to visualize the changes among the axial directions. The blue line, red line, orange line, and green line indicate the electric field intensity corresponding to 0.5, 0.2, $1/e^2$, and 0.1 times of the maximum at each depth.

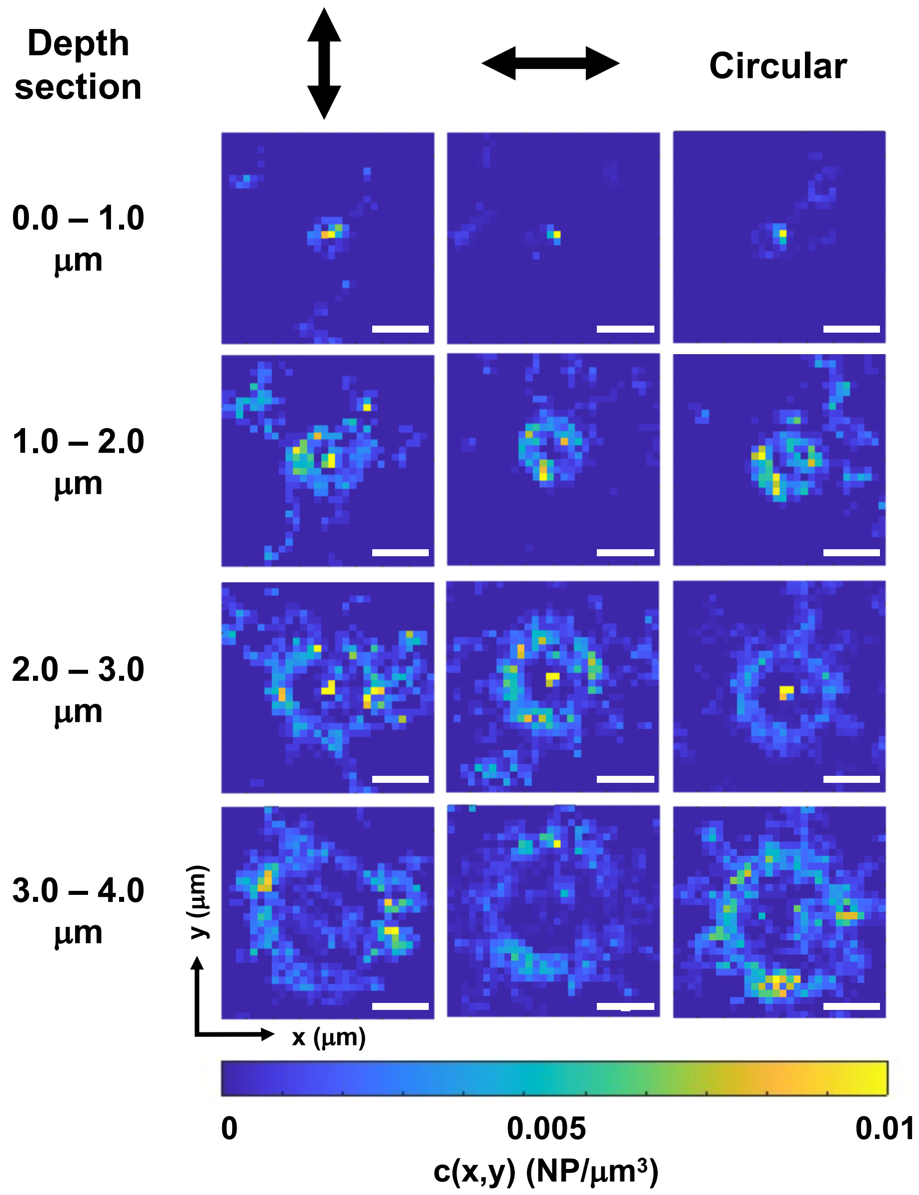

Figure S5 Average concentration field c(x,y) for three different laser polarizations (linear horizontal, lineal vertical, and circular) for different depths sections. The length of the scale bar is 2 µm.

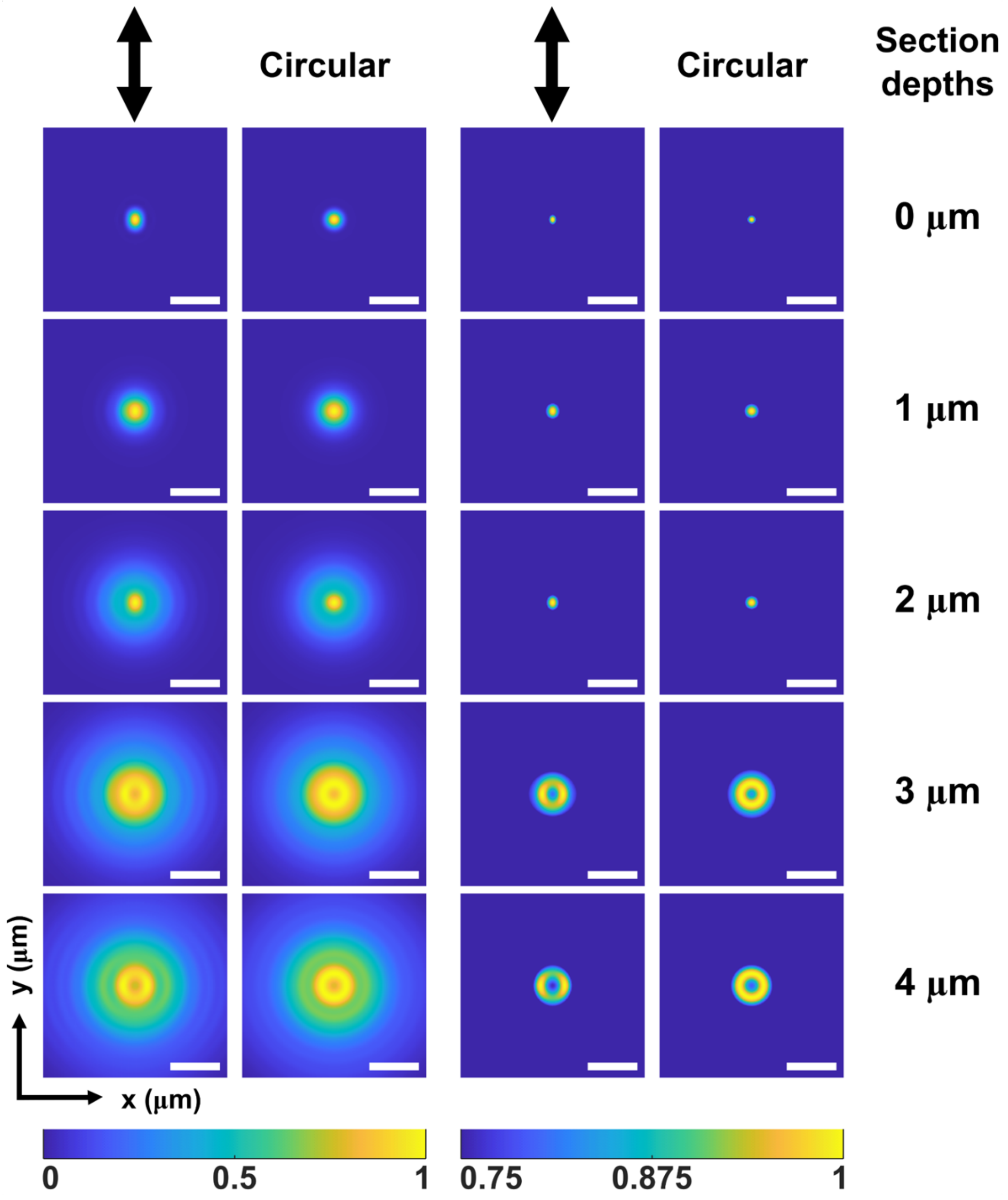

Figure S6 Calculated electric field intensity distribution for linear and circular polarization. In the right panel, we have saturated the color scale to observe the differences between the linear and the circular polarization. Specifically, the annular rings show a higher electric field intensity in the section parallel to the linear polarization, while a homogeneous distribution is observed for circular polarization. The electric field intensity is normalized at each depth. The white scale bar corresponds to 2 μm.

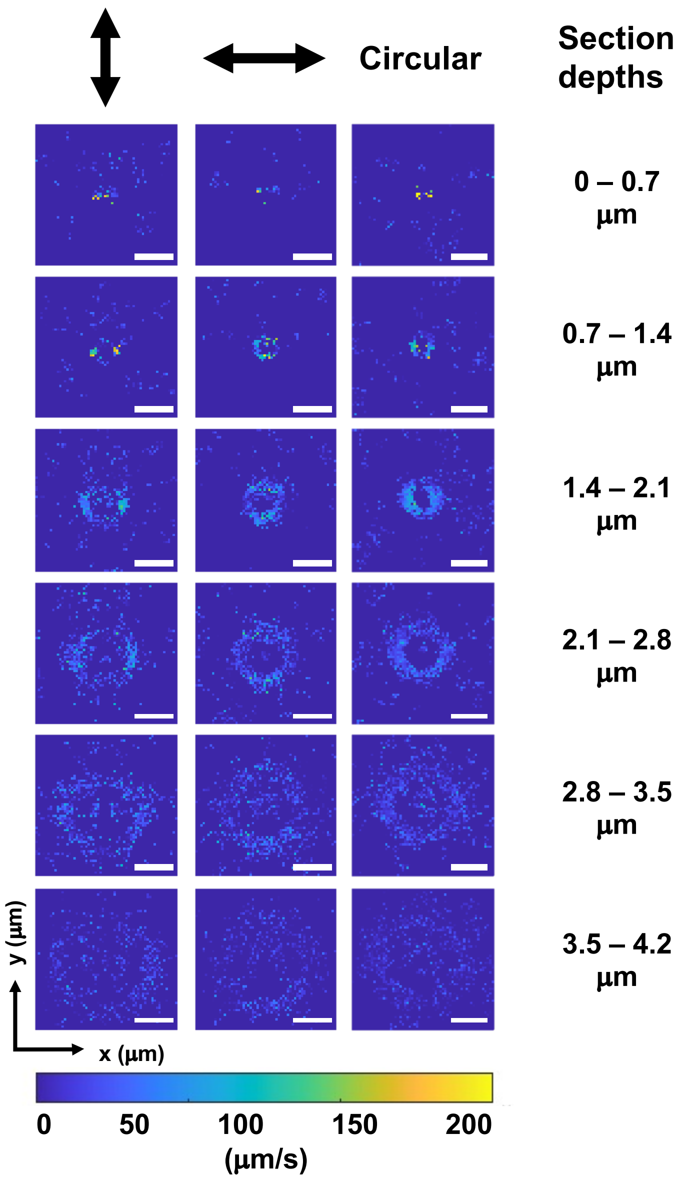

Figure S7 Effect of laser polarization on the 200 nm nanoparticle incorporation speed. Exactly, axial incorporation speed (V_z) distribution using a linear (horizontal and vertical) or circular laser polarization at different depth sections. We can observe that a homogeneous V_z speed distribution is obtained using a circularly polarized laser while a heterogeneous V_Z speed distribution is observed using linear polarized laser. In this case the region with a larger speed corresponds to the external annular ring section parallel to the direction of the linear laser polarization. The white scale bar corresponds to 2 µm. Each condition is obtained from 50 independent movies. The optical conditions are as follows: i) laser power after the objective 36 mW; ii) NA 1.20 (60x water-immersed objective).

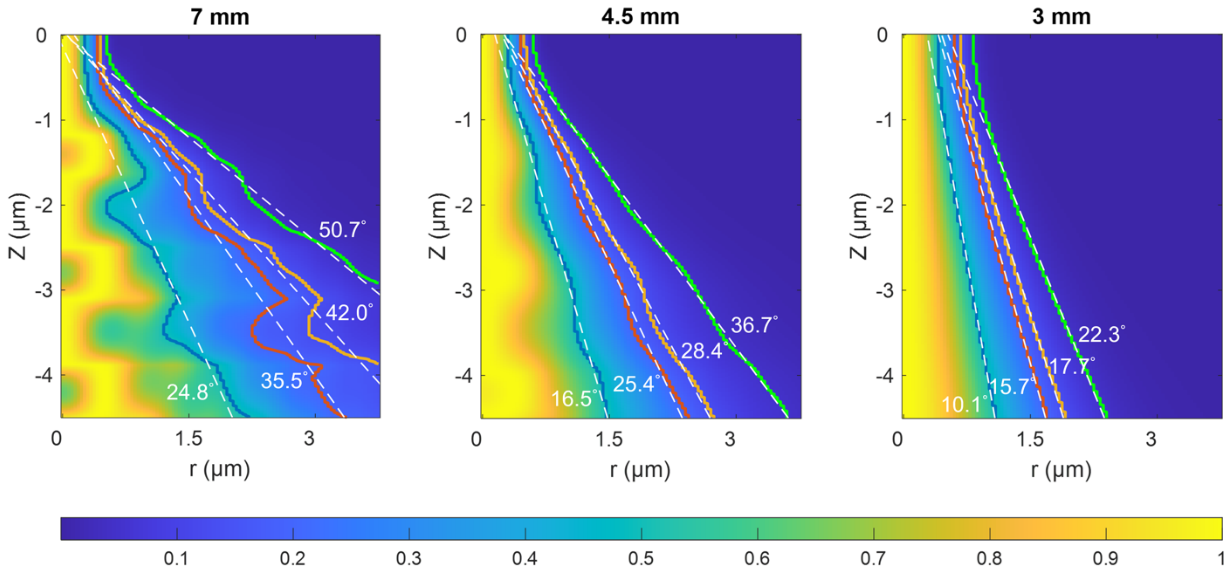

Figure S8 Side view of the calculated electric field intensity distribution for a tightly focused laser beam with circular polarization. The laser beam size before the objective lens was 7.0 mm (left), 4.5 mm (middle) and 3.0 (right). The intensity is normalized at each depth to visualize the changes among the axial directions. The blue line, red line, orange line, and green line indicate the electric field intensity corresponding to 0.5, 0.2, $1/e^2$, and 0.05 times of the maximum at each depth. The white dashed lines correspond to the estimated angle through the linear fitting of the contour defined by 50%, 20%, $1/e^2$ and 5% of the simulated electric field intensity for each laser beam diameter each electric field intensity.

Table S1 Experimental incorporation angle and the estimated angles corresponding to the contour defined by 50%, 20%, $1/e^2$ and 5% of the simulated electric field intensity for each laser beam diameter.

Laser Beam diameter	Experimental incorporation angle	0.5-fold electric field intensity	0.2-fold electric field intensity	$1/e^2$-fold electric field intensity	0.05-fold electric field intensity
7 mm	34°	24.8°	35.5°	42.0°	50.7°
4.5 mm	19°	16.5°	25.4°	28.4°	36.7°
3 mm	14°	10.1°	15.7°	17.7°	22.3°

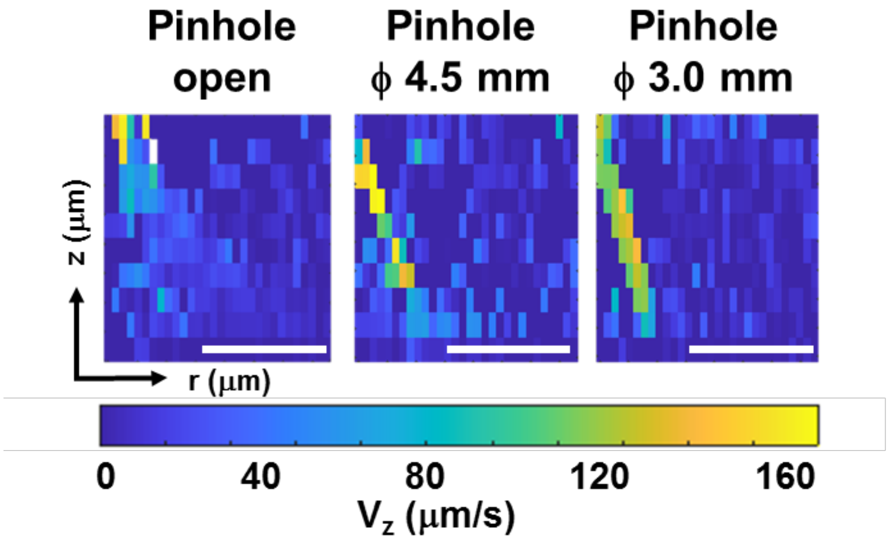

Figure S9 Effect of the effective numerical aperture on the 200 nm nanoparticle axial incorporation speed (V_z). The effective numerical aperture was controlled by changing the trapping laser beam size through a pinhole. The length of the scale bar is 2 μm.

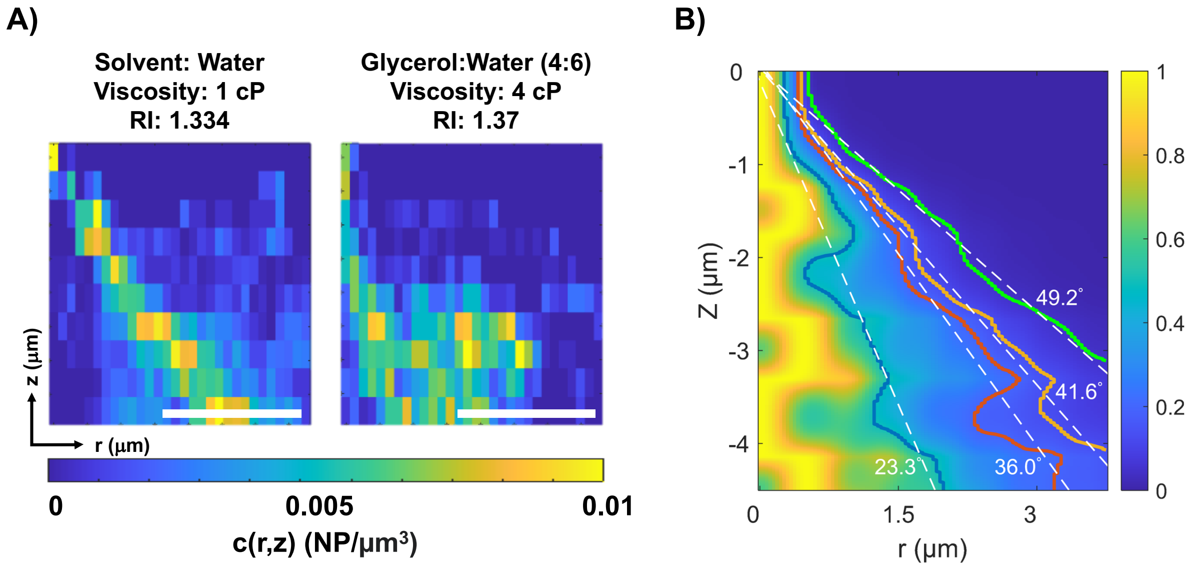

Figure S10 A): Average concentration field c(r,z) when the fluorescent NPs are suspended in two different solutions: left: neat water; right: glycerol/water mixture (4:6, w/w). The length of the scale bar is 2 µm. The viscosity and refractive index (RI) values of the mixture were obtained from reference [6]. B): Side view of the calculated electric field intensity distribution for a tightly focused laser beam with circular polarization. The laser beam size before the objective lens is 7.0 mm and the refractive index of the medium is 1.37 to mimic the glycerol/water (4:6 w/w) mixture. As in Figure S8, the white dashed lines correspond to the estimated angle through the linear fitting of the contour defined by 50%, 20%, $1/e^2$ and 5% of the simulated electric field intensity for each laser beam diameter.

Brief comment about Figure S10: when the refractive index increases from 1.334 to 1.37 in a glycerol:water (4:6 w/w) mixture, the calculated angle from the 20% contour slightly increases from 35.5° to 36.0°, while the experimental incorporation angle decreases to less than 8°. These two facts suggest that the incorporation angle is not only determined by the optical condition of the trapping laser beam, but also depends on other physicochemical factors such as viscosity. We hypothesize that the optical force is weaker than the increased solvent drag for the external channel in viscous environment. As result, the NPs are only incorporated through the internal channel where the electric field is the strongest and explains why the incorporation angle is reduced to less than 8°.

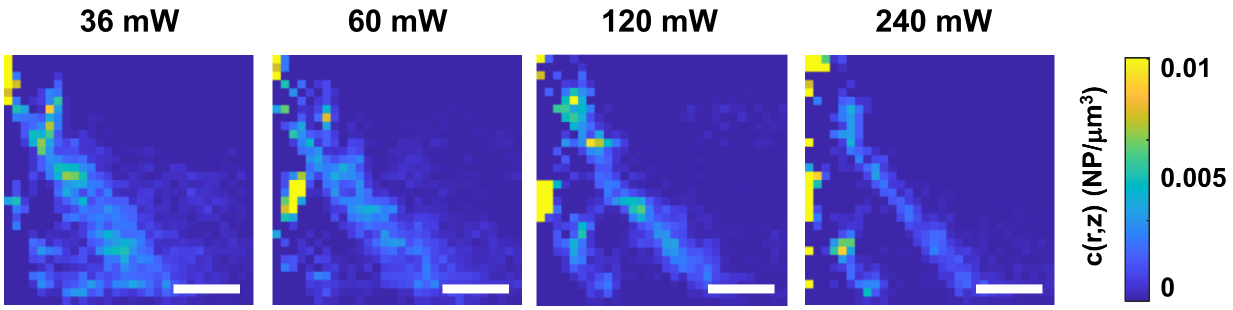

Figure S11. Effect of the 1064 nm trapping laser power (36, 60, 120 and 240 mW after the objective) on the NP concentration field (c(r,z))

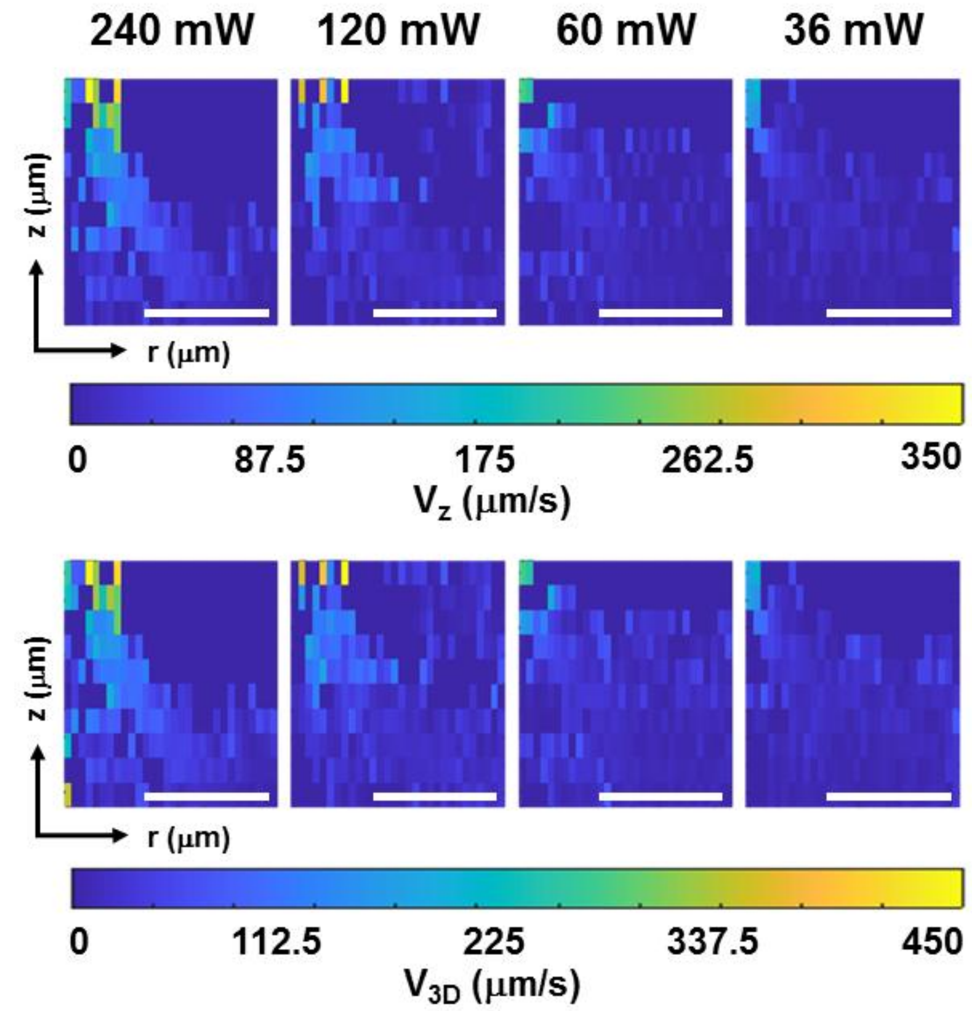

Figure S12. Effect of the 1064 nm trapping laser power (36, 60, 120 and 240 mW after the objective) on the 200 nm nanoparticle incorporation phenomenon. Top: Axial incorporation speed (V_z) distribution. Bottom: 3D incorporation speed (V_{3D}) distribution. The white scale bar corresponds to 2 μm.

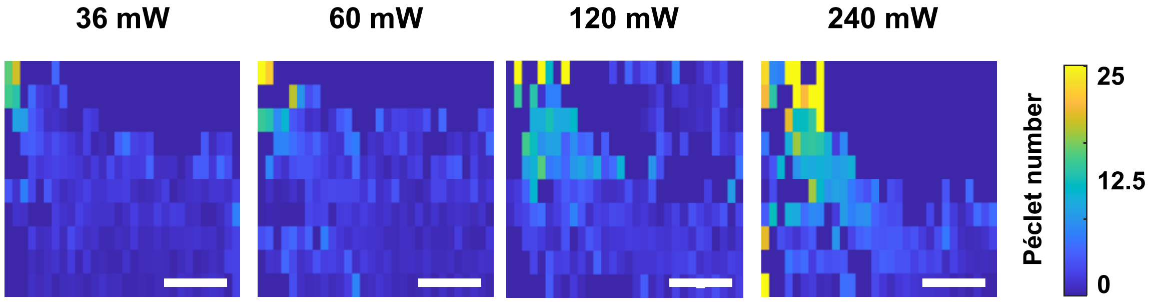

Figure S13. Effect of the 1064 nm trapping laser power (36, 60, 120 and 240 mW after the objective) on the Péclet number. The white scale bar corresponds to 2 μm.

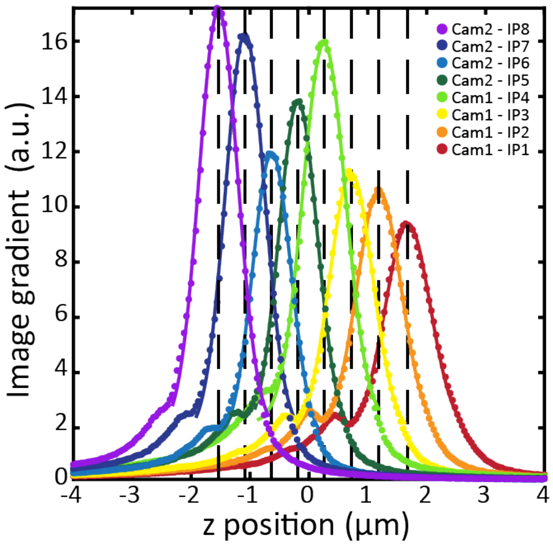

Fig. S14. To calibrate the position of the imaging planes, a sample consisting of fluorescent beads with a diameter of 0.2 micrometers was spin-cast onto a coverslip. This prepared reference sample was then scanned along the axial direction (z), with a step size of 50 nanometers, from a starting position of -4 micrometers to an ending position of +4 micrometers, in such a way that the fluorescent beads appeared to be in focus for each plane at some point in the sequence. The image gradient was calculated for each image and plane. The resulting data was plotted as a function of the axial position, as depicted in the chart. The image gradient displays a profile that is similar to a Gaussian distribution. By performing a Gaussian fit on the data, we were able to accurately determine the position of the imaging planes. Further details on this calibration process can be found in our previous technical note [1].

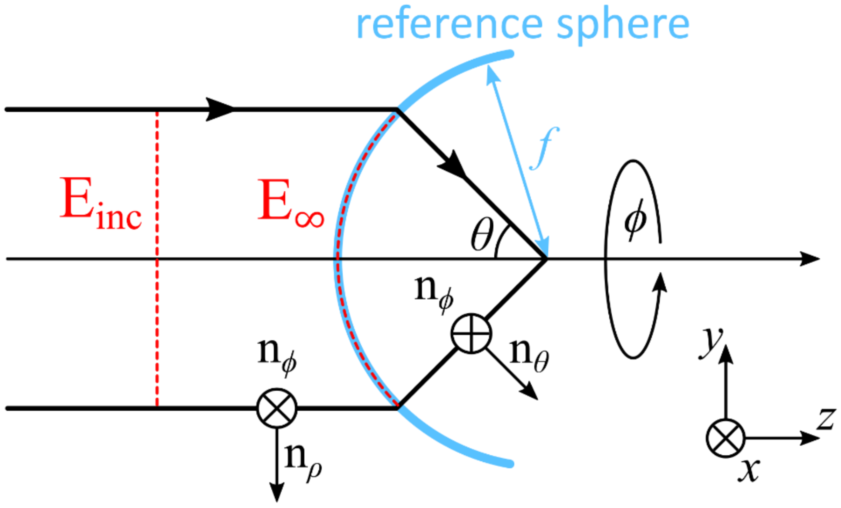

Fig. S15. The geometrical scheme of calculation model and the definition of its coordinates.[6] The reference sphere and the length (f) represent the aplanatic lens and its focal length, respectively. E_{inc} and E_∞ refer to the incident electric field and its projection on the reference sphere, respectively. n_ϕ and n_ρ are the unit vectors for describing s- and p-polarized field before refraction, while n_ϕ and n_θ are those after refraction. θ and ϕ are the angle for describing light focusing, their value should range from 0 to θ_{max} and 0 to 2 π , respectively.

Supplementary References

[1] B. Louis, R. Camacho, R. Bresolí-Obach, S. Abakumov, J. Vandaele, T. Kudo, H. Masuhara, I. G. Scheblykin, J. Hofkens, and S. Rocha, "Fast-tracking of single emitters in large volumes with nanometer precision," Opt. Express 28(19), 28656-28671 (2020).

[2] A. Sergé, N. Bertaux, H. Rigneault, and D. Marguet, "Dynamic multiple-target tracing to probe spatiotemporal cartography of cell membranes," Nat. Methods 5(8), 687–694 (2008).

[3] K. J. A. Martens, A. N. Bader, S. Baas, B. Rieger, and J. Hohlbein, "Phasor based single-molecule localization microscopy in 3D (pSMLM-3D): An algorithm for MHz localization rates using standard CPUs," J. Chem. Phys. 148(12), 123311 (2018).

[4] J. C. Crocker and D. G. Grier, "Methods of Digital Video Microscopy for Colloidal Studies," J. Colloid Interface Sci. 179(1), 298–310 (1996).

[5] J. Munkres, "Algorithms for the Assignment and Transportation Problems," J. Soc. Ind. Appl. Math. 5(1), 32–38 (1957).

[6] K. Takamura, H Fischer, N.R. Morrow, "Physical properties of aqueous glycerol solutions", J. Pet. Sci. Eng. 98–99, 50–60 (2012).

[7] Propagation and Focusing of Optical Fields. In Principles of Nano-Optics; Hecht, B., Novotny, L., Eds.; Cambridge University Press: Cambridge, 2006; 45–88.
